# A shared injury–homeostasis gradient in the human CSF proteome across neurological disease

**DOI:** 10.21203/rs.3.rs-10480838/v1

**Published:** 2026-08-11

**Authors:** Nicholas Mikolajewicz, Cecile Riviere-Cazaux, Kyle Tuohy, Sruthi Ranganathan, Rahul Kumar, Alexandra M. Miller, Manmeet S. Ahluwalia, Paul C. Boutros, Chetan Bettegowda, Terry C. Burns, Thomas Kislinger, Alireza Mansouri

**Affiliations:** 1 Peter Gilgan Centre for Research and Learning, Hospital for Sick Children, Toronto, ON, Canada; 2 Department of Molecular Genetics, University of Toronto, Toronto, ON, Canada; 3 Department of Neurosurgery, University of Toronto, Toronto, ON, Canada; 4 Department of Neurologic Surgery, Mayo Clinic, Rochester, Minnesota, USA; 5 Department of Neurosurgery, Penn State Milton S. Hershey Medical Center, Hershey, PA, USA; 6 Department of Medicine, University of Cambridge, Cambridge, UK; 7 Departments of Neurology and Pediatrics, Memorial Sloan Kettering Cancer Center, Manhattan, NY, USA; 8 Miami Cancer Institute, Baptist Health South Florida, Miami, Florida, USA; 9 Herbert Wertheim College of Medicine, Florida International University, Miami, FL, USA; 10 Sanford Burnham Prebys Medical Discovery Institute, La Jolla, California, USA; 11 Department of Neurosurgery, Johns Hopkins University School of Medicine, Baltimore, Maryland, USA; 12 Princess Margaret Cancer Centre, Toronto, ON, Canada

## Abstract

Cerebrospinal fluid (CSF) is the biofluid most proximal to the central nervous system (CNS), yet its proteomic organization across neurological disease remains unresolved. We assembled a meta-atlas of 35 CSF proteomic datasets (2016–2026) comprising 10,560 quality-controlled samples spanning neurodegenerative, neuroinflammatory, neuro-oncological, infectious and structural-injury contexts. Meta-analysis of cross-cohort detection defined a 747-protein core mass-spectrometry-accessible CSF proteome that generalizes to held-out cohorts. We derived thirteen consensus meta-programs that transfer across independent cohorts and span the major neural, barrier, immune and plasma compartments. Unexpectedly, these programs are dominated by a single axis of variation opposing complement- and plasma-associated inflammation against neuronal-synaptic homeostasis. This injury–homeostasis axis is diagnosis-spanning and tracks clinical severity and disease activity across diverse neurological conditions. We show that a two-protein C5–NRCAM model can approximate the injury–homeostasis axis, bridging deep proteomic profiling toward bedside assessment.

## 1 Introduction

Cerebrospinal fluid (CSF) is uniquely informative for the study of neurological disease because it is in direct continuity with the central nervous system and its barrier interfaces, such that its protein composition reflects both local CNS biology and regulated exchange with the circulation^1,2^. Deep proteomic studies have steadily expanded the detectable CSF proteome, from foundational catalogues of approximately 2,630 proteins to inventories exceeding 3,300 proteins^3–5^, while more recent disease-focused studies have shown that CSF proteomics can resolve clinically meaningful heterogeneity and biomarker panels in Alzheimer’s disease, dementia with Lewy bodies and multiple sclerosis^6–10^. Yet these advances have remained largely disease- and cohort-specific^11^. Consequently, it is still unclear which CSF proteins are reproducibly measurable across heterogeneous studies, which coordinated protein states are transferable between cohorts, and whether diverse neurological disorders share a common higher-order proteomic structure beyond diagnosis-specific signatures.

Here, we integrate cross-disease CSF proteomics from 35 cohorts to define a reproducible mass-spectrometry-accessible core proteome and to identify consensus programs of coordinated protein variation across neurological disease (Fig. 1). We show that these programs are organized by a dominant, disease-spanning injury–homeostasis axis that tracks clinical severity across CNS diseases and can be approximated by a two-protein C5–NRCAM model. By moving from single-disease marker discovery toward a transferable architecture of CSF proteomic states, this study establishes a generalizable framework and vocabulary for cross-cohort benchmarking and biological interpretation (https://nmikolajewicz.shinyapps.io/Axis/).

## 2 Results

### 2.1 Overview of CSF proteomics across neurological diseases

We assembled a meta-atlas of 35 CSF proteomic datasets (2016–2026, including two unpublished datasets), comprising 12,627 enrolled samples and 10,560 samples retained after per-study quality control (Fig. 1a, b, **Supplementary Table 1**). Studies span 15 countries, 11 disease types, and two proteomic platform families: mass spectrometry (MS; n=32, of which 2 used Seer Proteograph nanoparticle-enrichment MS) and affinity proteomics (proximity-extension assay [PEA] and aptamer; n=3). Per-study enrollment ranges from 10 to 7,008 samples with a median of 92 samples per study (IQR 40 – 150); observed protein depth ranges from 298 to 11,700. Of the 10,560 retained samples, 7,627 (72.2%) were assigned to one of eleven diagnostic themes (**Supplementary Table 2**) spanning reference controls (n=1,738), chronic demyelinating inflammation (n=2,020), high-grade neoplasms (n=1,113), chronic neurodegeneration/pain-fatigue (n=1,053), and seven additional acute and presymptomatic groups; the remaining 2,933 (27.8%) samples were retained as unassigned or mixed. CSF was sampled by lumbar puncture in 26 datasets, from the ventricular system in 7 (predominantly neuro-oncology cohorts; external ventricular drain [EVD]/Ommaya reservoirs), and mixed in two.

### 2.2 Definition and architecture of the core mass-spectrometry-accessible CSF proteome

Meta-analysis of protein detection across 21 discovery mass-spectrometry studies stratified 12,606 CSF proteins into five detection tiers (Tiers A-E; Supplementary Note 1, Extended Data Fig. 1, Extended Data Fig. 2a–d and **Supplementary Table 3**). From these we defined the 747-protein core, comprising 337 strict-core proteins [Tier A; lower bound of the 95% confidence interval (CI) for coverage-aware detection (CAD) rate ≥0.80] and 410 broad-recurrent proteins (Tier B; ≥0.50 to <0.80; Fig. 2a–c and **Supplementary Table 4**). A further 497 proteins were context-recurrent (Tier C; ≥0.20 to <0.50), 1,518 were not core (Tier D; <0.20), and 9,844 had insufficient support for classification (Tier E; fewer than six informative studies).

Core proteins were consistently detected regardless of lumbar (93.6% detection) or cranial (93.1%) CSF source (P = 0.926; Fig. 2d). In 12 held-out studies, Tier A proteins achieved a pooled area under the precision–recall curve (AUPRC) of 0.823 (95% CI, 0.812–0.840) and were detected in 77.5% of protein–study pairs (3,133 of 4,044; 95% CI, 76.2–78.8%; Fig. 2e). Together, these results support reproducible detection of the core proteome across independent studies and sampling sites.

Protein function, release mechanism and biological origin were associated with repeated detection (Fig. 2f–i). Plasma- and CNS/barrier-associated proteins, as well as secreted, complement/coagulation, glycoprotein and shed/ectodomain proteins, showed higher detection rates, whereas immune/myeloid-associated and intracellular proteins, particularly nuclear and mitochondrial proteins, were underrepresented (Fig. 2f, g). A Gaussian elastic-net model trained on the 2,762 Tier A–D proteins predicted observed detection rates (Spearman ρ = 0.543, 95% CI, 0.511–0.575; P < 2.2 × 10^−16^; Fig. 2h). The model predicts the 747 core proteins with an area under the receiver operating characteristic curve (AUROC) of 0.833 (95% CI, 0.815–0.849) and an AUPRC of 0.690 (95% CI, 0.658–0.721), outperforming the CSF probability model of Waury et al. on the same benchmark (ΔAUROC = 0.139, 95% CI, 0.115–0.161; ΔAUPRC = 0.258, 95% CI, 0.224–0.293; Fig. 2i and **Supplementary Table 5**).

The core proteome was biologically annotated through consensus co-clustering of latent-class probabilities derived from latent class modeling of protein function, release mechanism and origin features. This resolved six core classes (Fig. 2j and **Supplementary Table 6**): coagulation/complement (CC1; n = 190), glycosylation/proteostasis (CC2; n = 185), extracellular matrix (ECM)/matrisome (CC3; n = 92), neuronal adhesion/axonogenesis (CC4; n = 193), energy/NAD metabolism (CC5; n = 42) and TCA-cycle/mitochondrial respiration (CC6; n = 45) (Fig. 2k, Extended Data Fig. 2e–j and Extended Data Fig. 3). Latent class probabilities resolved three higher-order families in principal component space: ECM/barrier (CC1 and CC3), pan-CNS (CC2 and CC4) and metabolic/intracellular (CC5 and CC6) (Extended Data Fig. 2e–g). CC4 proteins were the most abundant in CSF and CC5–CC6 proteins the least (Kruskal–Wallis P = 7.0 × 10^−7^; Extended Data Fig. 2h). In external brain stable isotope labelling by amino acids in cell culture (SILAC) turnover data, CC3 proteins were the most stable (t_50_ = 383 h), whereas CC1 proteins had the shortest half-lives (t_50_ = 116 h; Kruskal–Wallis P = 1.77 × 10^−4^; Extended Data Fig. 2i)^12^.

Together, these analyses define a 747-protein, recurrently detectable and biologically structured core CSF proteome spanning barrier-associated, CNS-enriched and metabolic/intracellular biology.

### 2.3 Thirteen consensus meta-programs recover transferable proteomic states and variance structure in CSF

Having defined the recurrently detectable CSF proteome, we next examined coordinated variation in protein abundance. Benchmarking ten cross-cohort integration methods revealed a consistent trade-off between removing technical variation and preserving biological structure, limiting the use of globally corrected embeddings for direct biological discovery (Supplementary Note 2, Extended Data Fig. 4 and **Supplementary Table 7**). We therefore identified proteomic programs independently within each cohort and assessed their recurrence across cohorts.

Across 35 cohorts and three decomposition methods [non-negative matrix factorization (NMF), independent component analysis (ICA), and scale-free shared-nearest-neighbour network (SSN)], we identified 28,795 candidate programs. Filtering for within-study robustness, between-study recurrence and non-redundancy retained 634 programs, of which 247 clustered into 13 consensus meta-programs (Fig. 3a, b and **Supplementary Table 8**). Although defined independently of detection frequency, these meta-programs selectively overlapped the Tier A/B core proteome and Core Classes CC1–CC6, indicating that recurrent protein detection and coordinated abundance variation capture related but complementary properties of the CSF proteome (Supplementary Note 3 and Extended Data Fig. 5).

The 13 meta-programs generalized across independent cohorts (Extended Data Fig. 6a). In leave-one-study-out analyses, association weights learned from the training cohorts reconstructed held-out meta-program activity with a median Spearman’s ρ of 0.69 [interquartile range (IQR), 0.61–0.78], an AUROC of 0.97 [95% CI, 0.95–0.98] and an AUPRC of 0.67 (95% CI, 0.63–0.71). The median cross-cohort recovery was 94.3% at ρ ≥ 0.20 (Fig. 3c, Extended Data Fig. 6b–d and **Supplementary Table 9**), and protein-level meta-analytic correlations preserved the characteristic association structure of each program (Extended Data Fig. 6e). Ceiling-normalized recapitulation, which approximates the variance explained, yielded a median ratio of 0.61 (IQR, 0.41–0.71; sample-weighted mean, 0.78), indicating that the meta-programs capture a substantial fraction of variation in protein abundance within each cohort (Fig. 3d).

Functional annotation organized the meta-programs into six domains: (i) neuronal/synaptic programs, comprising MP8 (synaptic activity), MP5 (synaptic adhesion), MP2 (neural scaffold) and MP13 (synaptic junction); (ii) metabolic/catabolic and proteoglycan programs, comprising MP9 and MP11; (iii) ECM/stromal programs, comprising MP3; (iv) barrier/epithelial program, comprising MP7; (v) immune/inflammatory programs, comprising MP4 (myeloid stress), MP12 (neutrophil burst), MP10 (complement regulation) and MP6 (humoral immunity); and (vi) a plasma/complement program, MP1 (Fig. 3e, f; **Supplementary Table 10**). Orthogonal expression analyses in adult brain and fetal single-cell transcriptomic atlases supported these annotations, mapping neuronal/synaptic programs to neuronal populations, ECM/proteoglycan and barrier-associated programs to perivascular, choroid plexus/ependymal and glial compartments, immune programs to microglial, myeloid and lymphoid lineages, and plasma/complement features to blood- and liver-lineage expression (Extended Data Fig. 3; **Supplementary Table 11**). Independent plasma-ingress and frank-blood signatures showed selective, rather than global, enrichment (Supplementary Note 4, Extended Data Fig. 7 and **Supplementary Table 12**). Plasma ingress markers were enriched in MP1, with weaker associations with MP10, MP7, MP6 and MP12, whereas frank-blood signal was largely restricted to stress-associated MP4. The recurrence of plasma-derived proteins across diverse diagnostic groups positions plasma as a hallmark component of CSF rather than a contaminant.

These analyses establish a recurrent and generalizable set of meta-programs that capture a substantial fraction of proteomic variance in CSF and span its major biological domains.

### 2.4 A dominant injury–homeostasis axis organizes CSF meta-programs

CSF meta-program activities clustered into two antagonistic modules (Fig. 4a). Across 10,560 samples, the first principal component (PC1) captured this polarisation and explained 55.6% of meta-program-level variance (permutation P = 0.001; Fig. 4b). The second and third components described smaller axes (PC2, 13.7%; PC3, 10.7%) and enriched for ECM programs (Extended Data Fig. 8).

PC1 defines a CSF injury–homeostasis axis. The PC1-positive (injury) pole comprised 71 proteins (ρ ≥ 0.40, false discovery rate [FDR] < 5%; Fig. 4c) that were ubiquitously prevalent in CSF (detection rate 0.949; 95% CI, 0.927–0.971; range, 0.457–0.995) and enriched for complement/coagulation/plasma and inflammatory/immune processes (Fig. 4d–e, **Supplementary Tables 4 and 13**). Similarly, the PC1-negative (homeostatic) pole comprised 236 proteins (ρ ≤ −0.40, FDR < 5%) that were just as prevalent in CSF (detection rate 0.921; 95% CI, 0.913–0.930; range, 0.493–0.995) but enriched for neuronal adhesion and synaptic processes.

This loading structure was robust in sensitivity analyses. Re-deriving PC1 after weighting all datasets equally, and again after jointly excluding the Bader datasets^13^, preserved loading directions (minimum Tucker congruence coefficient = 0.998), indicating that the dominant axis was not an artefact of study size or of any single contributing study.

C5 and NRCAM were the strongest markers of the injury and homeostatic poles, respectively. Random-effects meta-analysis confirmed reciprocal associations for NRCAM (ρ = −0.71, 95% CI −0.76 to −0.64; 34 studies, n = 10,559) and C5 (ρ = 0.68, 95% CI 0.61 to 0.74; 31 studies, n = 10,438), with consistent directions across diagnoses (Extended Data Fig. 9).

Acute bacterial infections, structural injuries and high-grade neoplasms were positioned towards the injury pole; reference controls and presymptomatic or at-risk groups were positioned towards the homeostasis pole; and chronic inflammatory and neurodegenerative disorders occupied intermediate positions (Fig. 4f). Axis scores also correlated with the proportion of non-control profiles in mixed cohorts (Extended Data Fig. 10). The injury–homeostasis gradient therefore represents a dominant axis of CSF variation that spans across neurological conditions.

### 2.5 The injury–homeostasis axis tracks clinical severity across CNS disease

We next evaluated whether the CSF injury–homeostasis axis tracked clinically relevant outcomes. Higher axis scores were associated with treatment non-response, shorter overall survival and greater tumour burden in CNS lymphoma (Z_Stouffer_ = 4.78, FDR = 1.76 × 10^−6^; Fig. 4g and **Supplementary Table 14**); with leptomeningeal disease (LMD) positivity, progression and shorter survival (Z_Stouffer_ = 3.65, FDR = 0.0047; Fig. 4h); and with recurrence, WHO grade 4 rather than grade 2 disease, and shorter survival in glioma (Z_Stouffer_ = 5.14, FDR = 1.48 × 10^−6^; Fig. 4i). Neuronal-homeostatic features showed the inverse associations in LMD. In longitudinal glioma samples, axis activity increased early after resection (early versus preoperative: standardized mean difference (SMD) = 1.50, 95% CI 0.82–2.19; FDR = 1.4 × 10^−4^) and subsequently declined (delayed versus early: SMD = −1.71, 95% CI −2.56 to −0.86; FDR = 0.0020), indicating a dynamic injury response (Extended Data Fig. 11). Pan-oncology synthesis supported higher axis scores as a consistently adverse feature (Z_Stouffer_ = 3.77, FDR = 0.0015; Fig. 4j).

Beyond neuro-oncology, high-axis enrichment was observed across Alzheimer’s disease, dementia with Lewy bodies, frontotemporal dementia and Parkinson’s disease in an external cohort (Z_Stouffer_ = 7.14, FDR = 4.48 × 10^−18^; Fig. 4k). Axis scores were also higher in progressive than relapsing–remitting multiple sclerosis (Bader Astral: SMD = 0.37, 95% CI 0.19–0.55, FDR = 0.0020; TOF: SMD = 0.26, 95% CI 0.07–0.45, FDR = 0.0385), while remaining independent of oligoclonal-band status (SMD = −0.072, 95% CI −0.287 to 0.140; FDR = 0.715; Fig. 4l). Two normal-pressure hydrocephalus cohorts similarly showed an injury-biased state characterized by homeostatic disruption, ECM remodelling and increased MASP1 abundance (Extended Data Fig. 12a–g and **Supplementary Table 15**). Collectively, the axis captures shared inflammatory, tissue-injury and barrier-disruptive processes across diverse CNS diseases.

### 2.6 A C5–NRCAM model approximates the injury–homeostasis axis

We next show that C5 and NRCAM can be used to approximate the injury–homeostasis axis score. In the Riviere-Cazaux cohort, the affinity-based SomaScan assay provided separate measurements of intact C5, C5a, and C5b–6. Intact C5 alone showed moderate concordance with the axis score (R^2^ = 0.460, 95% CI 0.299–0.610). C5a performed similarly (R^2^ = 0.426, 95% CI 0.280–0.555), whereas combining intact C5, C5a and C5b–6 improved performance only marginally (R^2^ = 0.472, 95% CI 0.320–0.627). By contrast, combining NRCAM with intact C5 closely recapitulated the axis score (R^2^ = 0.869, 95% CI 0.811–0.900; Fig. 4m, **Supplementary Table 16**).

We evaluated this two-protein model in the larger and more heterogeneous Bader cohort and benchmarked it against routine CSF indices, including total protein, leukocyte count and the CSF-to-serum albumin quotient (QAlb). Linear models were trained using Astral data and evaluated without refitting in held-out timsTOF data. Total CSF protein and leukocyte count together recovered the axis with R^2^ = 0.430 (95% CI 0.411–0.449), which increased to 0.547 (95% CI 0.530–0.563) with the addition of QAlb. The C5–NRCAM model achieved R^2^ = 0.764 (95% CI 0.755–0.773), whereas combining both proteins with the three routine indices produced only a modest further improvement (R^2^ = 0.796, 95% CI 0.787–0.805; Fig. 4m,n). Across 34 cohorts, random-effects meta-analysis yielded a pooled R^2^ of 0.812 (95% CI 0.766–0.850; Fig. 4o and Supplementary Table 16).

Together, these findings nominate intact C5 and NRCAM as a candidate two-protein readout of the CSF injury–homeostasis axis. Low-axis samples retain neuronal-homeostatic features, whereas high-axis profiles have greater complement- and plasma-associated abundance, inflammatory features and injury-associated remodelling (Fig. 4p). This two-protein model provides a potential bridge between deep proteomic profiling and feasible bedside measurement of the axis.

## 3 Discussion

By integrating 35 cerebrospinal fluid (CSF) proteomic datasets comprising 10,560 quality-controlled samples, we define three complementary levels of CSF organization: a 747-protein recurrent mass-spectrometry-accessible core, 13 transferable meta-programs and a dominant, diagnosis-spanning injury–homeostasis axis. This establishes a shared proteomic structure across neurological diseases despite heterogeneity.

The 747-protein core defines recurrent measurability rather than an exhaustive inventory or healthy-state catalogue. Earlier CSF catalogues that identified 2,630–3,379 CSF proteins prioritized analytical depth in nominally normal cohorts^3–5^, whereas sequence-informed modelling predicted 3,189 proteins to be CSF-secreted at a >50% probability threshold^14^. We instead asked which proteins are consistently detected across heterogeneous studies. The core proteome (Tier A and B) provides a reproducibility floor for benchmarking and assay development, whereas lower-frequency tiers (Tier C and D) retain context-dependent and disease-associated candidates, including neurofilament light (NfL; *NEFL*) and tau (*MAPT*)^15–17^. The catalogue underrepresents low-abundance or transient mediators, including many cytokines such as interleukins, TNF and interferons^18^.

The 13 meta-programs are hallmark protein sets spanning neuronal–synaptic, barrier–matrix, metabolic–lysosomal, immune and complement-associated biology in CSF. They offer a universal vocabulary for comparing biology across cohorts and conditions, and a framework for reinterpreting existing biomarkers, such as GFAP in astrocytic injury^19^ or YWHAG in AD-associated cognitive decline^20^, both of which correlate with the MP4 (myeloid stress) meta-program in our cross-cohort meta-analysis (**Supplementary Table 13**).

The principal biological finding is a diagnosis-spanning injury–homeostasis axis, opposing a barrier–innate immunity state to neuronal-surface and synaptic homeostasis. This mirrors reactive-to-homeostatic gradients described in single-cell studies of glioblastoma^21^, astrocytes^22^, microglia^23^ and oligodendrocytes^24^. Concordant immune-barrier remodeling has also been observed in the ageing human hippocampus, where homeostatic microglia and barrier-supporting cells decline as pro-inflammatory myeloid states emerge^25^. The acute postoperative rise in the injury–homeostasis axis score followed by subsequent decline further supports its interpretation as a dynamic injury response. Consistent with this, acute infection, structural injury and high-grade neoplasia occupied the injury pole, whereas healthy controls and presymptomatic groups occupied the homeostatic pole. A liver- and QAlb-informed plasma signature enriched selectively for injury-associated MP1 and was near-orthogonal to a frank-blood signature, arguing against sample contamination as the sole explanation and instead supporting selective plasma ingress across altered CNS barriers. These plasma proteins are likely bioactive in the CNS; fibrinogen–CD11b/CD18 signalling promotes microglial clustering, axonal injury and synapse loss^26,27^, whereas C5aR1-positive microglia amplify neuroinflammation in acute-injury models^28^. Intact C5 is nevertheless best interpreted here as a sentinel of a complement-rich injury environment, not as direct evidence of C5 activation. Conversely, lower NRCAM and related neuronal-surface proteins may reflect altered neuronal integrity, proteolytic shedding or compositional dilution; these mechanisms cannot be distinguished here^29^.

C5 and NRCAM were the strongest opposing markers and together provided a compact surrogate for the injury–homeostasis axis. Within the Riviere-Cazaux cohort, the model showed R^2^ = 0.869; in held-out Bader timsTOF data, it achieved R^2^ = 0.764, compared with R^2^ = 0.547 for total protein, leukocyte count and QAlb. Across separately fitted cohort models, pooled R^2^ was 0.812. Because the target was a proteomic axis rather than an independent clinical outcome, C5–NRCAM should be viewed as a candidate two-protein readout requiring absolute quantification, cross-platform calibration and prospective demonstration of incremental clinical value. Its principal utility may lie not in diagnosis, but as a transdiagnostic state biomarker that positions patients along a continuum from neuronal homeostasis to active inflammatory injury. Analogous to C-reactive protein as a diagnosis-agnostic measure of systemic inflammatory burden, a validated C5–NRCAM assay could complement disease-specific CSF biomarkers, imaging, and routine indices to support risk stratification and longitudinal assessment of treatment response, postoperative recovery and disease progression.

Several limitations require consideration. Relative normalized abundances preclude inference about absolute concentration changes. Gene-symbol harmonization removed isoform-, proteoform- and cleavage-specific information, particularly for intact C5 versus C5a, and author-reported matrices inherited study-specific pre-processing. CSF collection site varied, with cranial sampling concentrated in neuro-oncology. Controls were heterogeneous, and age and sex were not systematically modelled due to a lack of consistent reporting. Study identification was goal-directed rather than exhaustive. The observational design cannot determine whether complement-associated injury drives neuronal-homeostatic loss or accompanies it. Within these constraints, the atlas provides a framework for reproducible measurement, cross-disease biological interpretation and compact readouts of shared CSF states.

## 4 Methods

### 4.1 Resources

#### Data availability.

Proteomic sample × protein matrices were obtained from source publications, supplementary files, or linked repositories; accession identifiers are provided in **Supplementary Table 1**. The Siletti et al. adult brain single-nucleus atlas was accessed through CELLxGENE (raw data: NEMO Archive, RRID: SCR_016152)^30^, and the Cao et al. fetal atlas (sci-RNA-seq3) from the Gene Expression Omnibus (GEO; GSE156793)^31^. The CSF meta-atlas is available through the companion web application (https://nmikolajewicz.shinyapps.io/Axis/).

#### Code availability.

Analysis code is available in the AXIS GitHub repository (https://github.com/NMikolajewicz/Mikolajewicz-2026-CSF-MetaAtlas) under the MIT licence and is archived at Zenodo (DOI: https://doi.org/10.5281/zenodo.21608891).

### 4.2 Computing environment

#### Software.

Analyses were performed in R 4.5.2, and figures were assembled in CorelDRAW X8.

#### Computational specifications.

Analyses were run on Windows 10 Pro (v21H2) using an Intel Core i9-10900K processor (3.70 GHz; 10 cores/20 threads) and 128 GB RAM.

### 4.3 Cohort assembly and preprocessing

#### Search strategy and inclusion criteria.

Studies were identified on February 24, 2026, through structured searches of PubMed and ProteomeXchange (PRIDE and MassIVE), reference-chaining from CSF proteomics reviews and biomarker meta-analyses, and the authors’ curated library. Search strings combined “cerebrospinal fluid” with proteomics terms (“mass spectrometry”, “LC-MS/MS”, “Olink”, “SomaScan”, “proteomics”, “TMT”, “DIA”, and “DDA”) and disease terms spanning neuro-oncology, neurodegeneration, neuroinflammation, neuroinfection, trauma, and chronic pain. Eligible studies included human CSF analysed by discovery-scale proteomics, cohort size > 5, and an available sample × protein abundance matrix; CSF sampling method was unrestricted. The search was goal-directed rather than exhaustive. The final meta-atlas comprised 35 datasets^13,15,17,32–59^.

#### Metadata curation.

For each study, we recorded first author, year, DOI, geographic origin, cohort design, diagnostic groups and sample sizes, age and sex by group, CSF sampling site, and proteomic platform, instrument, and acquisition mode (**Supplementary Table 1**).

#### Per-study matrix preprocessing.

Sample × protein matrices were mapped to UniProt gene symbols, with duplicate identifiers averaged. Samples with > 80% missing values were excluded; proteins required < 90% missingness and at least two observations. Zeros were treated as missing in mass-spectrometry datasets and as observed in affinity assays. Dataset scale was inferred from its empirical distribution: raw count or intensity matrices were log_2_-transformed, 0–1 matrices were logit-transformed, and already logged, fold-change, or z-scaled matrices were retained. Samples were median-centred and divided by the median absolute deviation. Missing values were imputed within datasets using missRanger v2.4.0 (predictive mean matching; pmm.k = 5, 100 trees, maxiter = 3, max.depth = 6, seed = 42), selected based on label-free proteomics benchmarks^60^. Scaled, imputed matrices were used for cross-study correction; non-imputed matrices were used for core-proteome analysis.

#### Matrix-correction benchmarking.

Nine correction methods were benchmarked against the uncorrected matrix, yielding ten matrix states on a shared 917-protein × 10,560-sample matrix: ComBat, limma, quantile normalization, TAMPOR, WaveICA, Scanorama, and BERT (Batch-Effect-Reduction-Tree) with ComBat, limma, or TAMPOR inner models^61–64^. Study was the batch variable.

Performance was summarized across three batch-removal metrics: local k-nearest-neighbour batch-composition test (k = 15, Euclidean)^65^, sign-inverted batch average silhouette width, and principal-component-regression batch reduction, and six biology-preservation metrics: concordance of cohort-level Wilcoxon differential-abundance statistics before versus after correction, Jaccard overlap of highly variable proteins, diagnosis k-nearest-neighbour balanced accuracy (k = 15; stratified cross-validation), diagnosis average silhouette width, diagnosis-group graph connectivity, and isolated-label average silhouette width. Metrics were oriented so that higher values indicated better performance and min–max scaled within domains; the composite was 0.4 × batch removal + 0.6 × biology preservation. The limma-corrected 917-protein matrix was used for atlas-wide sample- and protein-level analyses, with BERT-limma providing a complementary 3,000-protein matrix (**Supplementary Table 7**; Extended Data Fig. 4).

### 4.4 The mass-spectrometry-accessible core CSF proteome

#### Coverage-aware assayable universe.

Core-proteome analysis used 21 mass-spectrometry datasets that retained informative missingness (mean protein missingness > 2% or interquartile range > 5%). Studies were ordered by observed protein depth. For each study, a protein was considered assayable if detected in that study or in at least two depth-matched studies within a centred three-study window. Coverage-aware protein detection was then calculated as the within-study detection proportion among studies in which the protein was assayable (**Supplementary Table 3**).

#### Tiered CSF proteome derivation.

Study-level logit-transformed detection proportions were pooled by random-effects meta-analysis, and tiers were assigned from the lower 95% confidence bound: Tier A ≥ 0.80, Tier B ≥ 0.50 to < 0.80, Tier C ≥ 0.20 to < 0.50, and Tier D < 0.20. Leave-one-study-out analyses reassigned proteins that crossed a tier boundary in > 20% of fits by majority rule; proteins supported by fewer than six informative studies were assigned to Tier E. Tiers A and B defined the 747-protein core. Perturbing cutoffs by 2.5–5% yielded Jaccard agreement of 0.82–0.96 denoting relative stability across tier thresholds.

#### External validation of the core CSF proteome.

Generalization was assessed in 12 held-out studies using the pooled lower 95% confidence bound as the prediction score and observed detection as the outcome. The same score was benchmarked against the Waury high-confidence CSF reference (probability ≥ 0.90)^14^. Performance was reported by AUROC and AUPRC.

#### Core-proteome annotation.

Core proteins were annotated with 64 features (**Supplementary Table 6**) spanning: (i) tissue or cell origin from the Human Protein Atlas^66^, JensenLab^67^, CellMarker 2.0^68^, and PanglaoDB^69^; (ii) subcellular localization from DeepLoc 2.1^70^, OmniPath^71^, MatrixDB^72^, and the matrisome^73^; and (iii) release mechanism from SignalP 6.0^74^, DeepSMP^75^, and SheddomeDB^76^.

#### Detection model.

An elastic-net Gaussian model (glmnet v4.1–10; α and λ selected jointly by five-fold cross-validation) predicted detection rate for 2,762 Tier A–D proteins from the 64 annotations (**Supplementary Table 5**). Predicted and observed rates were compared by Spearman ρ. On the shared protein universe, this model and the Waury probability score were compared for Tier A/B versus Tier C/D discrimination using AUROC and AUPRC; 95% confidence intervals and between-model differences were bootstrapped.

#### Core Class derivation: latent-class modelling and consensus clustering.

Latent structure among the 747 core proteins was modelled with VarSelLCM v2.1.3.2 over K = 3–12^77^. Across random restarts, 40 of 64 features were stably selected (mean Jaccard = 0.98); no CSF abundance or meta-atlas features were retained, making the partitions independent of abundance, batch, and diagnosis. Because restart concordance was moderate (mean adjusted Rand index = 0.43), final class assignments used consensus clustering. Gower distances on the selected features were decomposed by principal coordinates analysis with Cailliez correction, retaining dimensions that explained ≥ 90% of variance. VarSelLCM was then refit 100 times to random 85% column subsamples of the retained coordinates across K = 3–12. The resulting 747 × 747 co-clustering matrix was clustered with Ward’s D^2^ linkage; the cluster count minimizing the proportion of ambiguous clustering yielded CC1–CC6. Primary and secondary assignments were the two highest posterior weights, and confidence was the maximum posterior probability.

#### Core class characterization.

Within each sample, core class abundance was the median of corrected abundance z scores across member proteins. Classes were compared across 10,560 paired profiles by Kruskal–Wallis tests followed by paired Wilcoxon signed-rank contrasts. SILAC half-lives for 232 mapped proteins were compared by Kruskal–Wallis and pairwise Wilcoxon tests^12^.

### 4.5 Recurrent CSF proteomic meta-programs

#### Within-study component-program discovery.

Candidate component programs were derived independently within each study after retaining proteins detected in ≥ 50% of samples and in at least two studies. Each matrix was decomposed by non-negative matrix factorization (NMF), independent component analysis (ICA), and a scale-free shared-nearest-neighbour (SSN) graph.

NMF (NNLM v0.4.4, nnmf) used mean-squared-error (MSE) loss on (i) a shifted matrix, X − min(X), and (ii) a sign-split matrix formed by row-concatenating X^+^ = max(X, 0) and X^−^ = max(−X, 0). For each rank K = 3–15, 10 fits were run (≤500 iterations; relative tolerance 10^−5^), retaining the lowest-MSE solution. ICA (ica v1.0.3, icafast) used the same rank grid and fitting parameters without non-negative transformation. For each NMF or ICA component, the 50 highest-loading proteins from each loading-vector pole defined candidate programs. For SSN (scMiko v1.0, runSSN)^78^, protein profiles were reduced by principal component analysis (PCA; 90% variance; < 30 PCs), embedded in a shared-nearest-neighbour graph, and Louvain-clustered at resolutions 0.2–1.2 (step 0.1). Proteins below the 10th percentile of within-cluster edge weight were removed; the remainder were ranked by a composite score (C_SSN_) combining min–max-normalized intramodular edge weight (w_SSN_) and within-cluster Spearman correlation (ρ_SSN_):

$$C_{SSN}=0.7\times w_{SSN}+0.3\times \rho_{SSN}$$

The top 50 CSSN-ranked proteins defined each study- and resolution-specific SSN program.

#### Robustness filtering and deduplication.

Candidate programs from NMF, ICA and SSN discovery step were filtered sequentially^79^: within-study robustness required ≥ 30 of 50 proteins shared with at least two programs at another rank (NMF or ICA) or resolution (SSN); between-study robustness required ≥ 10 of 50 proteins shared with a program from another study; and greedy within-study deduplication removed programs sharing ≥ 10 proteins with a higher-ranked program, where ranking reflected aggregate cross-study overlap. These filters reduced 28,795 candidates to 10,805 robust and then 634 non-redundant programs.

#### Consensus meta-program assembly.

The 634 programs were grouped by iterative community detection on a graph linking top-50 sets that shared ≥ 10 proteins^79^. The most-connected unassigned program with at least three qualifying neighbours and its highest-overlap partner seeded each community. Additional programs joined if they shared ≥ 10 proteins with the current consensus top-50 set, which was updated after each addition by majority vote and next-rank replenishment. Communities were excluded if > 30% of proteins matched ribosomal or mitochondrial symbols (RPL|RPS|MRPL|MRPS|MT) or if fewer than two studies contributed. Thirteen consensus meta-programs remained (MP1–MP13; **Supplementary Table 8**).

#### Transferability.

Transferability was tested by leave-one-study-out prediction across 35 cohorts. For each held-out study, protein–program Spearman correlations were estimated in the remaining cohorts. Up to 250 proteins with the largest absolute non-zero weights were intersected with the held-out feature set, and weights were normalized by their absolute sum. Predicted activity was the weighted sum of z-scored protein abundance. Recovery was the Spearman correlation between predicted and observed activity; ρ ≥ 0.20 defined recovery (**Supplementary Table 9**). The recovery operating point was defined as the Spearman correlation at which the precision and false-positive rate curves intersected (Extended Data Fig. 6b). For each evaluable cohort × meta-program pair, 200 null correlations were generated from independent Gaussian vectors matched to cohort sample size. AUROC and AUPRC quantified separation of observed from null correlations.

#### Null-standardized variance explained.

To test whether meta-programs captured more abundance structure than random protein sets, we analysed 33 cohorts with adequate coverage (excluding Spreafico 2017 and Timirci-Kahraman 2019). Protein matrices were column-centred, program activity was the mean z-scored abundance of member proteins, and the centred matrix was reconstructed by ridge regression with nested cross-validation (leave-one-out for n ≤ 100; otherwise five-fold). Within each training fold, λ minimized MSE in a three-fold inner split over 10^−3^–10^3^. Cross-validated fraction of variance explained was:

$$\text{FVE}=1-\frac{\|Y-\hat{Y}{\|}_{F}^{2}}{\|Y{\|}_{F}^{2}}=1-\frac{\text{prediction error}}{\text{total variance}}$$

where Y is the centred protein matrix, Ŷ its out-of-fold reconstruction, and ‖·‖F the Frobenius norm. The procedure was repeated with 100 size-matched random protein sets per program. Observed FVE was standardized against the cohort-specific null and pooled across cohorts by random-effects meta-analysis.

#### PCA-ceiling recapitulation.

The recapitulation ratio, *R*_*i*_ = FVE_*MP*_/FVE_*PC*_, compared meta-program FVE with the variance explained by an in-sample 13-component PCA and was clamped to [0,1].

#### Protein–program associations.

Within each study with at least eight complete samples, Spearman correlations between protein abundance and gene-set variation analysis (GSVA) v1.50 meta-program activity were computed for every protein–program pair and pooled by Fisher-z random-effects meta-analysis. P values were Benjamini–Hochberg (BH)-adjusted within programs (**Supplementary Table 13**).

### 4.6 Plasma and blood signatures

#### Signature derivation.

Two signatures were derived: a 20-protein plasma-ingress signature reflecting blood–CSF barrier permeability and a 10-protein frank-blood signature reflecting sample contamination. Plasma candidates required (i) higher abundance in plasma than CSF^80,81^, (ii) liver specificity in HPA RNA consensus and JensenLab, and (iii) association with the CSF-to-serum albumin quotient (QAlb). Liver specificity required log_2_(liver/highest non-liver) ≥ 2.0, τ ≥ 0.95, and Gini ≥ 0.85. Within studies with at least 10 complete samples, protein abundance was correlated with log_10_(QAlb), and Spearman estimates were pooled by Fisher-z random-effects meta-analysis^13,40,51^. Erythrocyte- and platelet-associated proteins in PanglaoDB, CellMarker 2.0, JensenLab, or HPA blood resources were excluded. Frank-blood candidates were derived from bloody-versus-clean CSF contrasts^13,46,49^; study-level log_2_ fold changes were pooled by inverse-variance random-effects meta-analysis and intersected with a priori erythrocyte markers from the same resources (**Supplementary Table 12**).

#### Sample-level scoring and meta-program coupling.

GSVA v1.50 generated sample-level plasma-ingress and frank-blood scores. Within-study Spearman correlations with the 13 meta-program scores were pooled by Fisher-z random-effects meta-analysis.

### 4.7 Injury–homeostasis axis derivation

#### Axis derivation and characterization.

GSVA v1.50 was used to score the 13 meta-programs in 10,560 samples from the limma-corrected 917-protein matrix. Scores were centred and scaled before principal component analysis, and PC1 was retained as the injury–homeostasis axis. PC1-PC13 significance was tested against 1,000 column-wise permutations; leave-one-study-out resampling assessed stability (mean pairwise inter-study ρ = 0.93). PC1 loadings were additionally re-derived after weighting all datasets equally and after jointly excluding the Bader datasets, with congruence to the primary solution quantified by Tucker’s congruence coefficient. Within-cohort meta-program correlations were Fisher-z pooled across studies, and the pooled matrix was clustered by Ward’s D^2^ on 1 − ρ to define opposing modules.

#### Protein-level axis association.

Within each cohort, protein abundance was correlated with axis score for proteins with at least eight complete observations; correlations were Fisher-z pooled and BH-adjusted. Axis-positive and axis-negative sets required |ρ| ≥ 0.40 and FDR ≤ 0.05 (**Supplementary Tables 4 and 13**). A protein-set surrogate was derived from GSVA scores for the two sets and compared with PC1 by Spearman correlation. For axis score prediction models, C5 and NRCAM were removed from the scoring sets to prevent predictor leakage. Pole markers were ranked by pooled ρ; forest plots used inverse-variance weights and 95% confidence intervals.

#### Clinical associations across CNS disease contexts.

Axis and meta-program scores were tested against multiple clinical endpoints in CNS lymphoma, leptomeningeal disease, glioma, neurodegenerative disease (Alzheimer’s disease, dementia with Lewy bodies, frontotemporal dementia, and Parkinson’s disease)^82^, and neuroinflammatory cohorts. Binary outcomes used logistic regression, continuous outcomes used Spearman correlation or linear models, and time-to-event outcomes used Cox proportional-hazards regression (**Supplementary Table 14**); group contrasts were summarized by Cohen’s d. The neurodegenerative cohort (AD n = 1,883; DLB n = 763; FTD n = 772; PD n = 1,465) was excluded from discovery and evaluated using aggregate differential-abundance statistics.

The signed magnitude of each test statistic was direction-normalized so that positive values denoted worse outcomes (death, progression, higher grade, or greater burden), defining the Clinical Outcome Index. Endpoint-level evidence across CNS lymphoma, leptomeningeal disease, and glioma was combined by the weighted Stouffer Z method^83^.

In neuroinflammatory cohorts, standardized mean differences compared progressive versus relapsing–remitting multiple sclerosis and oligoclonal-band-positive versus -negative CSF.

#### Longitudinal glioma analysis.

Longitudinal analyses used the Riviere-Cazaux 2025 glioma cohort. Resection-epoch analysis included 53 samples from 22 patients: pre-operative (n = 22), early post-operative (≤14 days; n = 20), and delayed post-operative (>14 days; n = 11). Therapy-state analysis included primary (n = 53), adjuvant temozolomide (n = 4), post-chemoradiation (n = 5), and recurrent disease (n = 37).

Both analyses profiled 18 features: MP1–MP13, axis score, C5, NRCAM, and plasma-ingress and frank-blood scores. Group differences used Kruskal–Wallis tests; standardized mean profiles were clustered with Ward’s D^2^ on Euclidean distance. Paired axis trajectories were plotted across resection epochs.

#### Axis-score prediction models.

Linear models tested whether C5–NRCAM and routine CSF measures approximated the continuous axis. The Riviere-Cazaux 2025 cohort compared intact C5, C5a, the C5b–6 complex, and combinations with NRCAM; the Bader 2026 cohort compared C5/NRCAM with total CSF protein, leukocyte count, and QAlb. Models were trained in the Bader Astral subset (n = 1,627 complete cases) and evaluated without refitting in pooled timsTOF subsets (n = 4,854); complete cases were used without imputation. The outcome was the eigenscore of GSVA enrichment for axis-positive and axis-negative protein sets, with C5 and NRCAM removed. Quantitative predictors were log_2_-transformed. Held-out performance was reported as RMSE, R^2^, and Spearman ρ.

#### NPH differential abundance and classifier.

Adult normal-pressure hydrocephalus differential abundance was estimated with limma in the Mikolajewicz cohort (20 NPH versus 1,747 pooled atlas controls) and the de Geus cohort (44 NPH versus 53 study-matched controls)^39,46^. Replicated proteins required FDR ≤ 0.05 and |log_2_FC| ≥ 0.30 in both cohorts with concordant direction.

A pooled classifier distinguished 64 adult NPH samples from 5,771 other atlas samples using class-weighted logistic regression (glmnet v4.1–10) with stratified five-fold cross-validation. The operating threshold maximized Youden’s J on pooled held-out predictions. Models used MASP1 alone or a five-protein panel (MASP1, LTBP2, RNASE4, ENO2, L1CAM; **Supplementary Table 15**).

### 4.8 Protein set annotation and characterization

#### Hypergeometric over-representation analysis (ORA).

Set-level enrichment used the Bader Lab collections (GO, Reactome, KEGG, Hallmark, and NABA matrisome). Backgrounds were analysis-specific: the 747 core proteins for CC1–CC6, and the 15,880-protein measured union for MP1–MP13 (**Supplementary Table 10**) and the plasma-ingress and frank-blood signatures. Significant meta-program terms were grouped by pairwise Jaccard overlap into six domains: neuronal/synaptic, metabolic/catabolic, ECM/stromal, barrier/epithelial, immune/inflammatory, and plasma/complement.

#### Axis-ranked gene-set enrichment analysis.

Proteins ranked by pooled axis ρ were tested by pre-ranked GSEA against Gene Ontology Biological Process, Reactome, and MSigDB Hallmark sets. Terms with FDR ≤ 0.05 were connected by Jaccard overlap and embedded as an enrichment network; communities were labelled by dominant biological theme.

#### Protein-class enrichment.

Categorical enrichments used Fisher’s exact tests. Core Classes were tested for origin, release-mechanism, and compartment annotations. For detectability analyses, the highest and lowest quintiles of observed detection among 2,762 proteins (n = 553 each) were compared for tissue/cell-type and protein-class enrichment.

#### Single-cell projection onto reference atlases.

Feature sets (MP1–MP13, CC1–CC6, axis-positive and axis-negative signatures, plasma-ingress and frank-blood signatures, C5, and NRCAM) were projected onto the Siletti et al. (2023) adult brain atlas (3,369,219 nuclei; 106 dissections; three donors) and the Cao et al. (2020) fetal atlas (4,062,980 nuclei; 121 samples from 28 fetuses, 72–129 days post-conception; 15 organs). UMAPs were used for visualization. Seurat v5.4.0 AddModuleScore generated feature-set scores; C5 and NRCAM were shown as z-scored expression. Cell-type mean scores and expression were displayed as heatmaps with rows and columns clustered by Euclidean distance and complete linkage (**Supplementary Table 11**).

### 4.9 Statistical analysis

Unless stated otherwise, tests were two-sided with α = 0.05 and Benjamini–Hochberg FDR control. Cross-cohort estimates used restricted maximum likelihood (REML) random-effects meta-analysis with Hartung–Knapp–Sidik–Jonkman 95% confidence intervals; Spearman correlations were Fisher-z transformed, proportions were logit-transformed, and heterogeneity was summarized by τ^2^ and I^2^. Z scores were combined by Stouffer’s method where indicated. Two-group contrasts used Wilcoxon rank-sum tests; comparisons of three or more groups used Kruskal–Wallis tests with BH-adjusted post-hoc contrasts. Cohort-level differential abundance used limma. Predictive performance was estimated from held-out stratified cross-validation and summarized by AUROC and AUPRC with bootstrap confidence intervals.

## Extended Data

**Extended Data Fig. 1.**
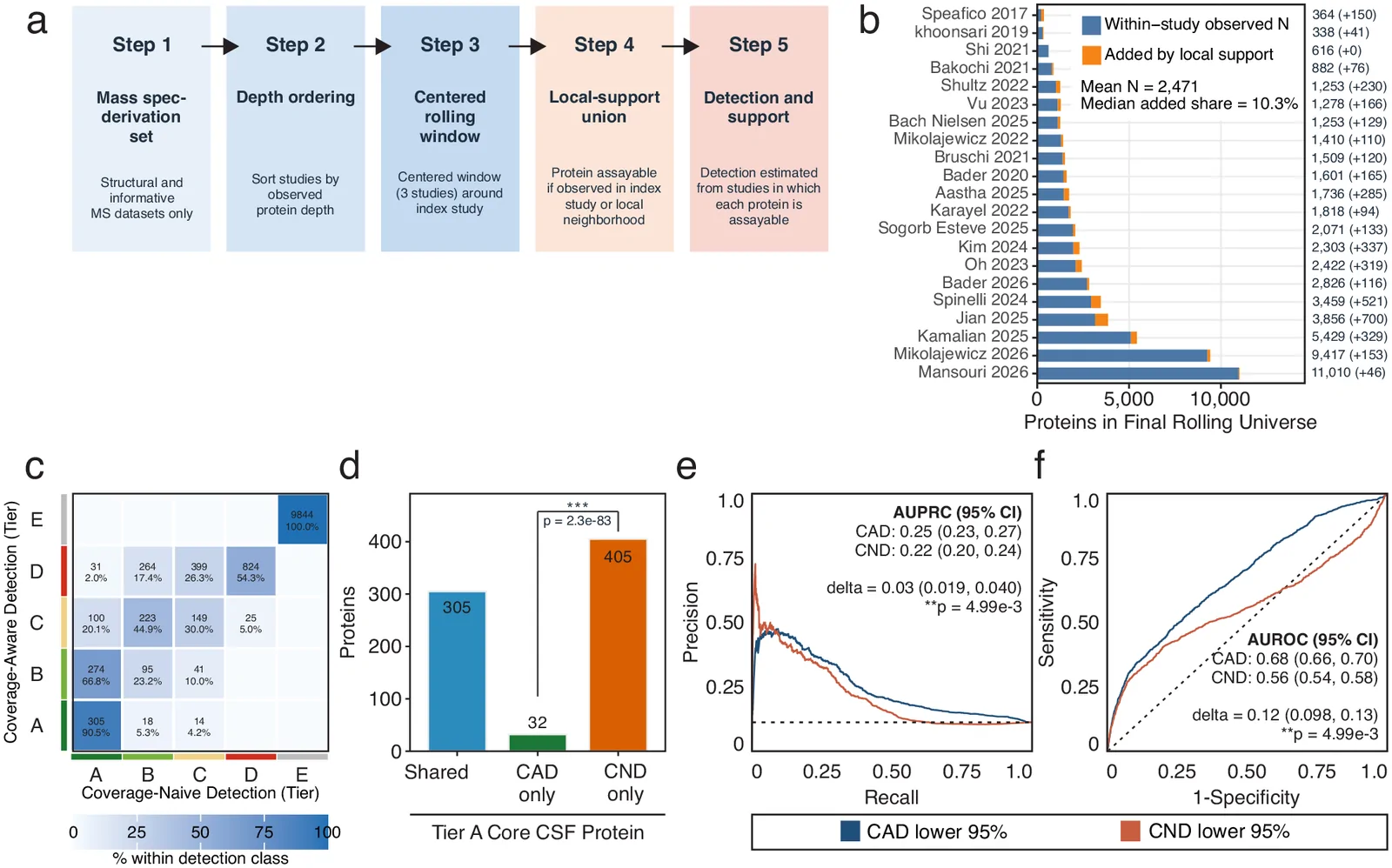
Coverage-aware definition and validation of the assayable CSF proteome. **(a)** Coverage-aware detection workflow. Twenty-one informative mass-spectrometry (MS) studies were ordered by protein depth. For each index study, the assayable universe comprised observed proteins plus proteins supported by depth-matched studies within a centred three-study window. Detection tiers were assigned from the pooled lower 95% confidence bound, with proteins supported by fewer than six informative studies assigned to Tier E. **(b)** Assayable protein universes across the 21 derivation studies, ordered by observed depth. *Blue*, observed proteins; *orange*, proteins added by local support. **(c)** Correspondence between coverage-aware and coverage-naive detection-tier assignments. Tiles show protein counts and row-normalized percentages. **(d)** Overlap between Tier A proteins identified by CAD and CND-based approach. **(e,f)** Precision–recall (**e**) and receiver operating characteristic (**f**) benchmarks of CAD and CND scores against the mapped Waury *et al*. high-confidence CSF reference (probability ≥ 0.90; *n* = 12,021 proteins)^14^. CAD, coverage-aware detection; CND, coverage-naive detection.

**Extended Data Fig. 2.**
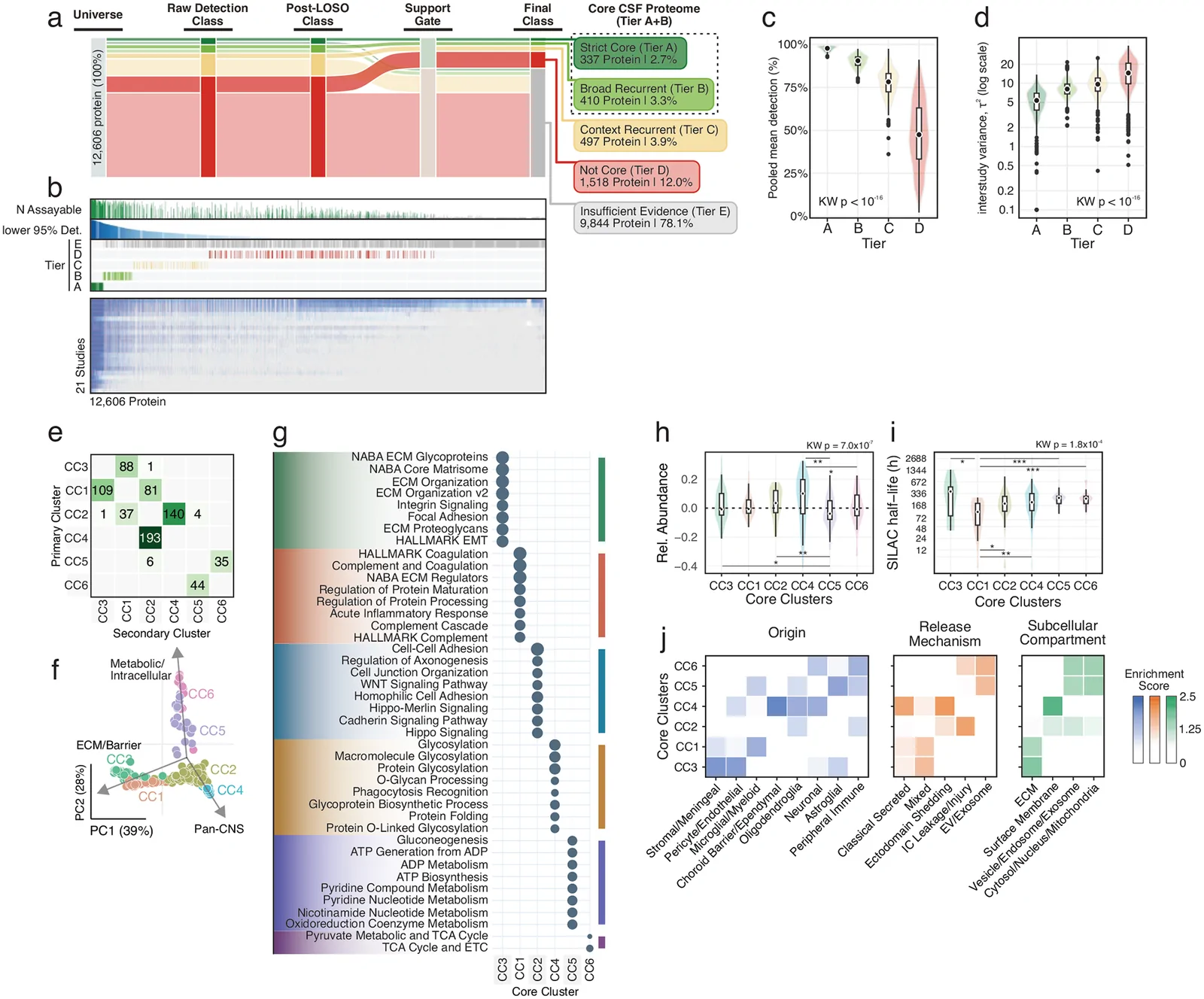
Detection-tier architecture and six biological classes of the core CSF proteome. **(a)** Classification of 12,606 proteins across 21 informative MS studies through initial coverage-aware tiering, leave-one-study-out reassignment, minimum-support filtering and final tier assignment. **(b)** Protein-by-study detection matrix. Proteins are ordered by the pooled lower 95% confidence bound and studies by proteomic depth; *marginal tracks*, number of informative studies, pooled lower 95% bound and final tier. **(c,d)** Pooled CAD estimates (**c**) and between-study heterogeneity, τ^2^ (**d**; logarithmic scale), across Tiers A–D. *Violin plots*, distributions, *boxes*, median and IQR (Kruskal–Wallis: **c**, *P* < 2.2 × 10^−16^; **d**, *P* = 1.86 × 10^−208^). **(e)** Cross-tabulation of primary and secondary Core Class assignments among the 747 Tier A/B proteins. **(f)** PCA of the 747 proteins in latent-class probability space. *Points*, protein; *colour*, core class (CC1–CC6). PC1 and PC2 separate extracellular-matrix (ECM)/barrier, pan-CNS and metabolic/intracellular families. **(g)** Representative pathways enriched in each core class. **(h)** Sample-level core class abundance across 10,560 paired CSF profiles. Kruskal–Wallis *P* = 7.0 × 10^−7^. **(i)** Brain-protein half-lives for 232 mapped proteins in external stable-isotope-labelling-by-amino-acids-in-cell-culture (SILAC) data. Kruskal–Wallis *P* = 1.8 × 10^−4^. (**j)** Enrichment of core classes by protein origin, release mechanism and subcellular compartment. *Fill*, Fisher’s-exact-test enrichment score.

**Extended Data Fig. 3.**
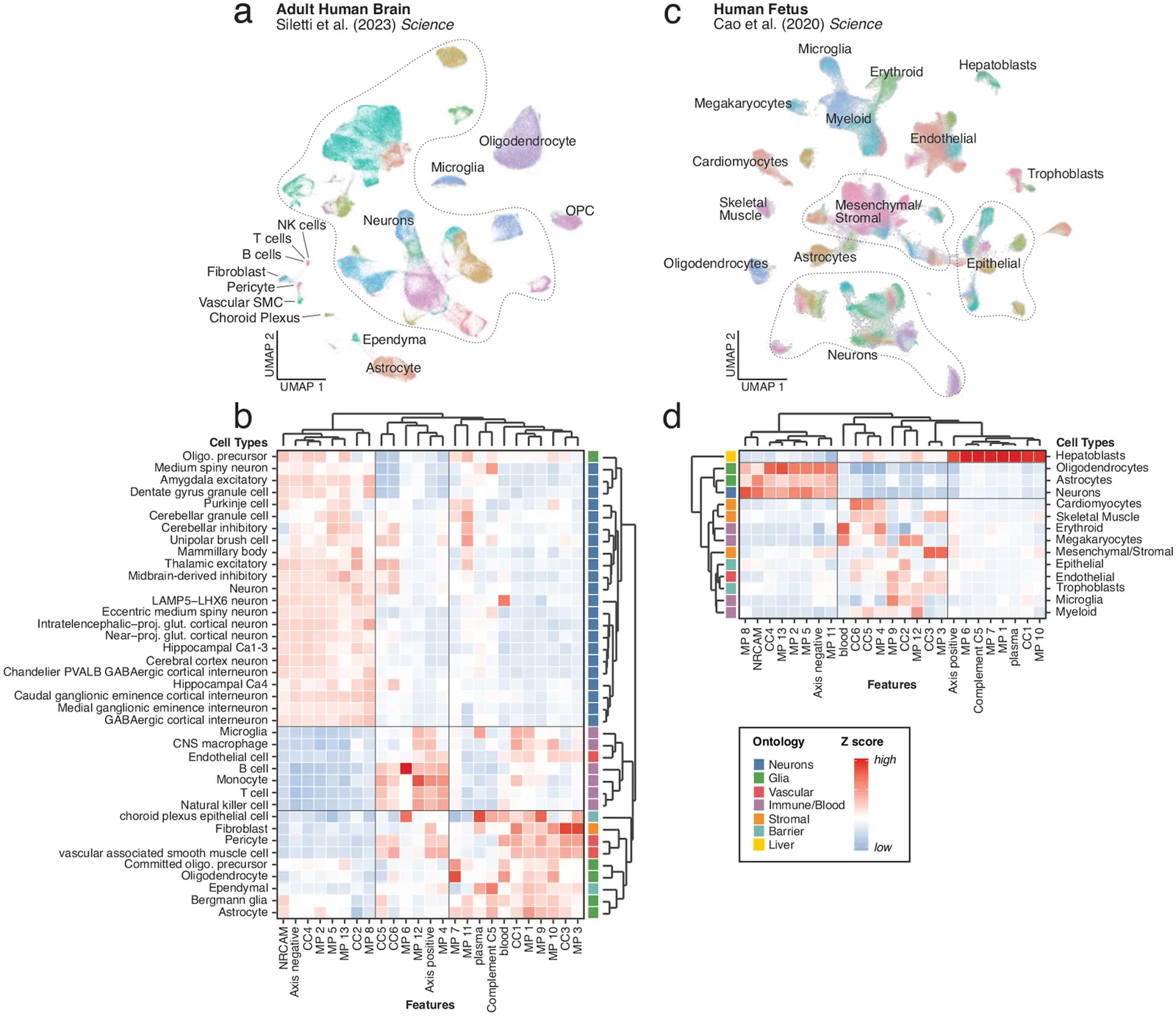
Single-cell transcriptomic atlases localize CSF proteomic programs. **(a)** UMAP of the adult human brain single-nucleus RNA-sequencing atlas (approximately 3 million nuclei from 106 dissections and three donors)^30^. *Dashed outlines*, broad neuronal populations. **(b)** Cell-type expression of CSF meta-programs (MP1–MP13), core classes (CC1–CC6), injury- and homeostasis-axis signatures, plasma-ingress and frank-blood signatures, and the sentinel proteins C5 and NRCAM. *Fill*, z-scored mean module scores, or z-scored expression for individual proteins; rows and columns were hierarchically clustered. **(c)** UMAP of the human fetal cell atlas (approximately 4 million cells across 15 organs)^31^. *Dashed outlines*, major lineage compartments. **(d)** Lineage-level expression of the same CSF feature sets, scaled and clustered as in **b**.

**Extended Data Fig. 4.**
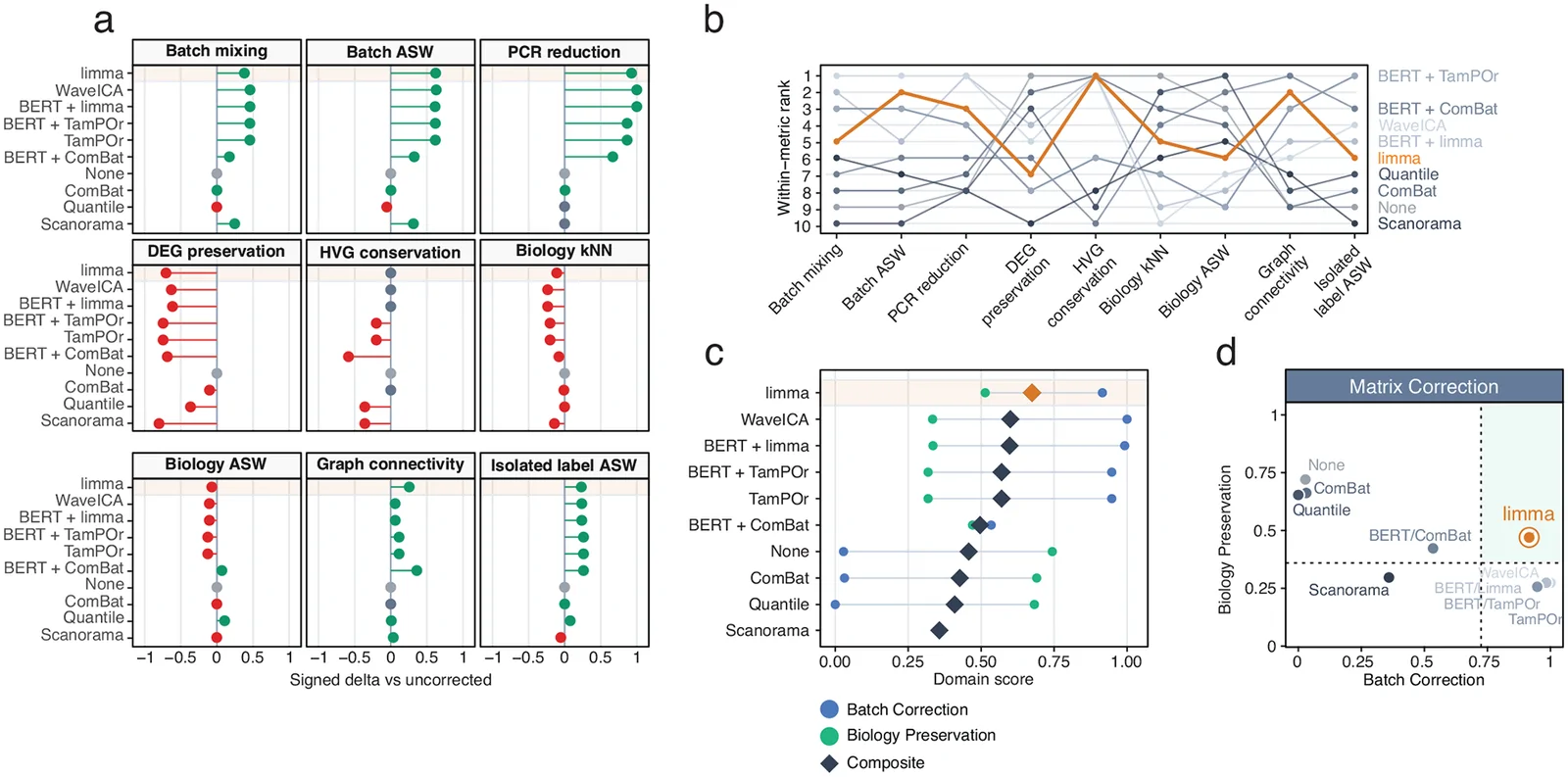
Benchmarking matrix-correction methods identifies limma as the best balance of batch removal and biological preservation. **(a)** Signed changes relative to the uncorrected 917-protein matrix across nine metrics and ten correction methods. Positive values indicate improvement; batch average silhouette width (ASW) was sign-reversed so that higher values uniformly denote better performance. Metrics assessed batch removal (batch mixing, batch ASW and principal-component-regression batch reduction) or biological preservation (differential-abundance preservation, highly variable feature conservation, biology *k*-nearest-neighbour accuracy, biology ASW, graph connectivity and isolated-label ASW). **(b)** Direction-aware within-metric ranks, with rank 1 indicating the best-performing method. **(c)** Batch-removal, biological-preservation and composite scores. The composite score weighted biological preservation more strongly (0.4 × batch score + 0.6 × biology score). **(d)** Joint comparison of batch-removal and biological-preservation scores, highlighting limma as the most balanced method.

**Extended Data Fig. 5.**
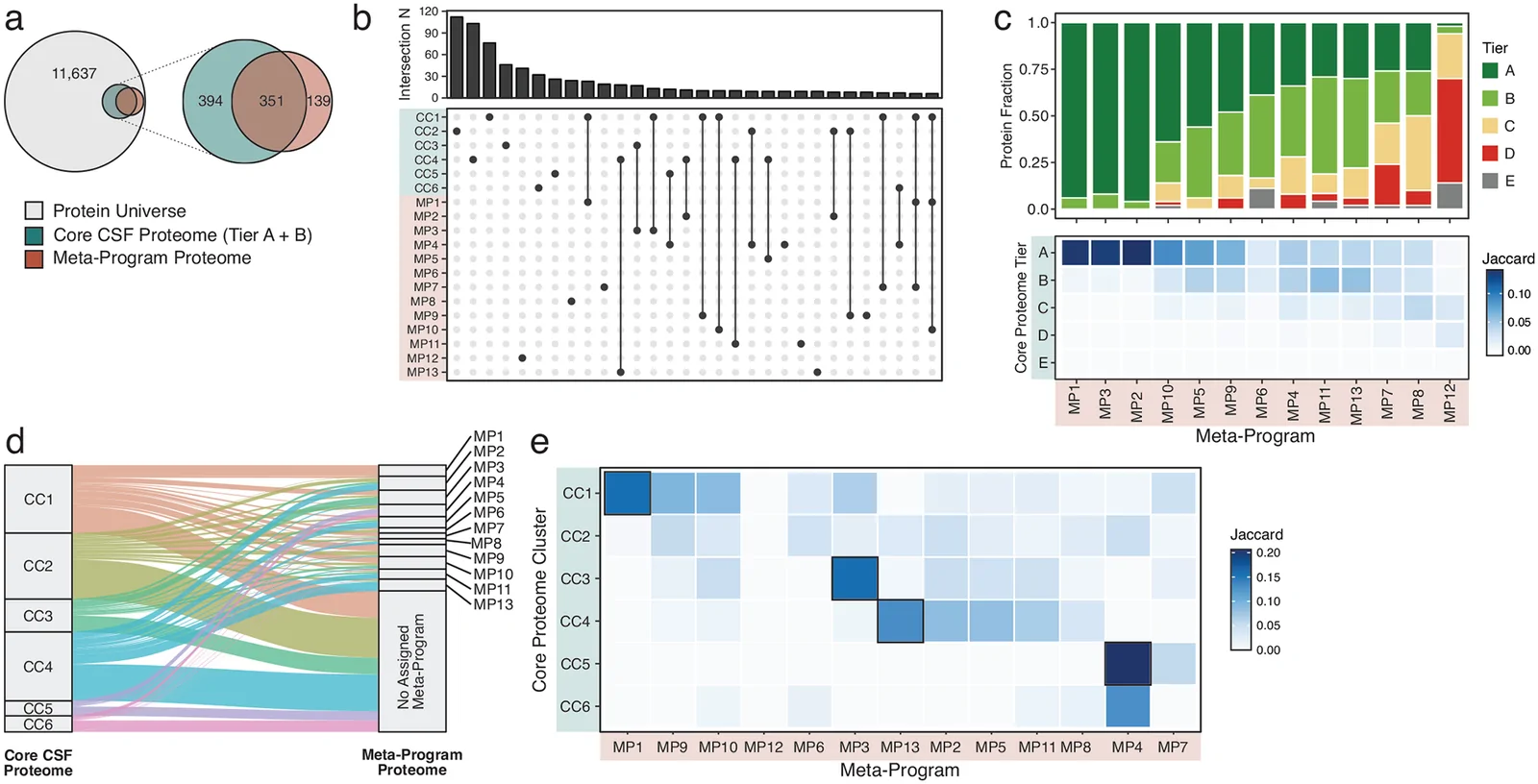
Overlap between core CSF proteome clusters and meta-programs. **(a)** Euler diagram of the detectable CSF proteome, the 747-protein core CSF proteome (Tier A/B), and the union of the 13 consensus meta-program (MP1–MP13) protein sets. **(b)** UpSet plot of multi-membership intersections across the 6 core classes (CC1–CC6) and the 13 meta-programs. **(c)** Jaccard similarity between core tier and meta-programs (*bottom*) and tier composition of each meta-program (*top*). **(d)** Alluvial diagram linking the Core Class proteins to their resolved meta-program assignments. **(e)** Jaccard similarity between core classes (CC1–CC6) and meta-programs. Outlined cells mark reciprocal best matches.

**Extended Data Fig. 6.**
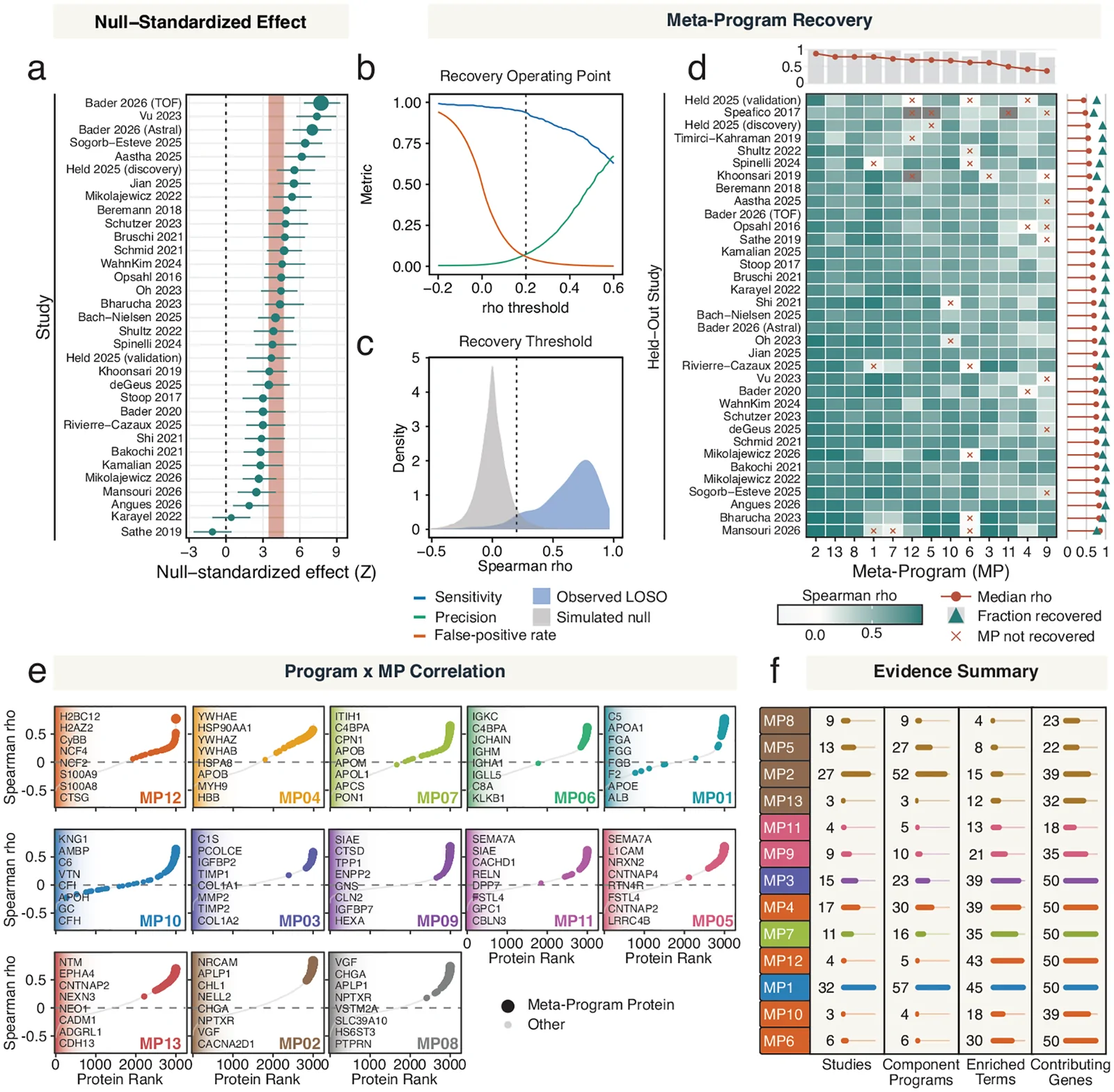
Cross-cohort recovery and protein-level support of CSF proteomic meta-programs. **(a)** Null-standardized variance capture by cohort compared with size-matched random protein sets. *Points*, cohort-level z-scores with 95% CI; *point size*, cohort size; *shaded band*, random-effects pooled estimate. **(b)** Threshold operating curves for leave-one-study-out (LOSO) correlations treated as observed positives and sample-size-matched random correlations treated as negatives. *Dashed line*, prespecified threshold Spearman ρ = 0.20. **(c)** Density distributions for the same observed and random correlations. **(d)** Leave-one-study-out transferability across 35 held-out cohorts. The heatmap shows the Spearman correlation (ρ) between predicted and observed meta-program activity for each held-out study–meta-program pair. *Red crosses* indicate failure to meet the prespecified recovery threshold (ρ < 0.20). *Marginal summaries* show median correlation and fraction recovered. **(e)** Proteins rank-ordered by random-effects pooled Spearman correlation with meta-program activity. *Coloured points*, meta-program member proteins. **(f)** Meta-program evidence comprising contributing discovery studies, assigned component programs, enriched terms, and contributing genes. Bar lengths are scaled separately within each column.

**Extended Data Fig. 7.**
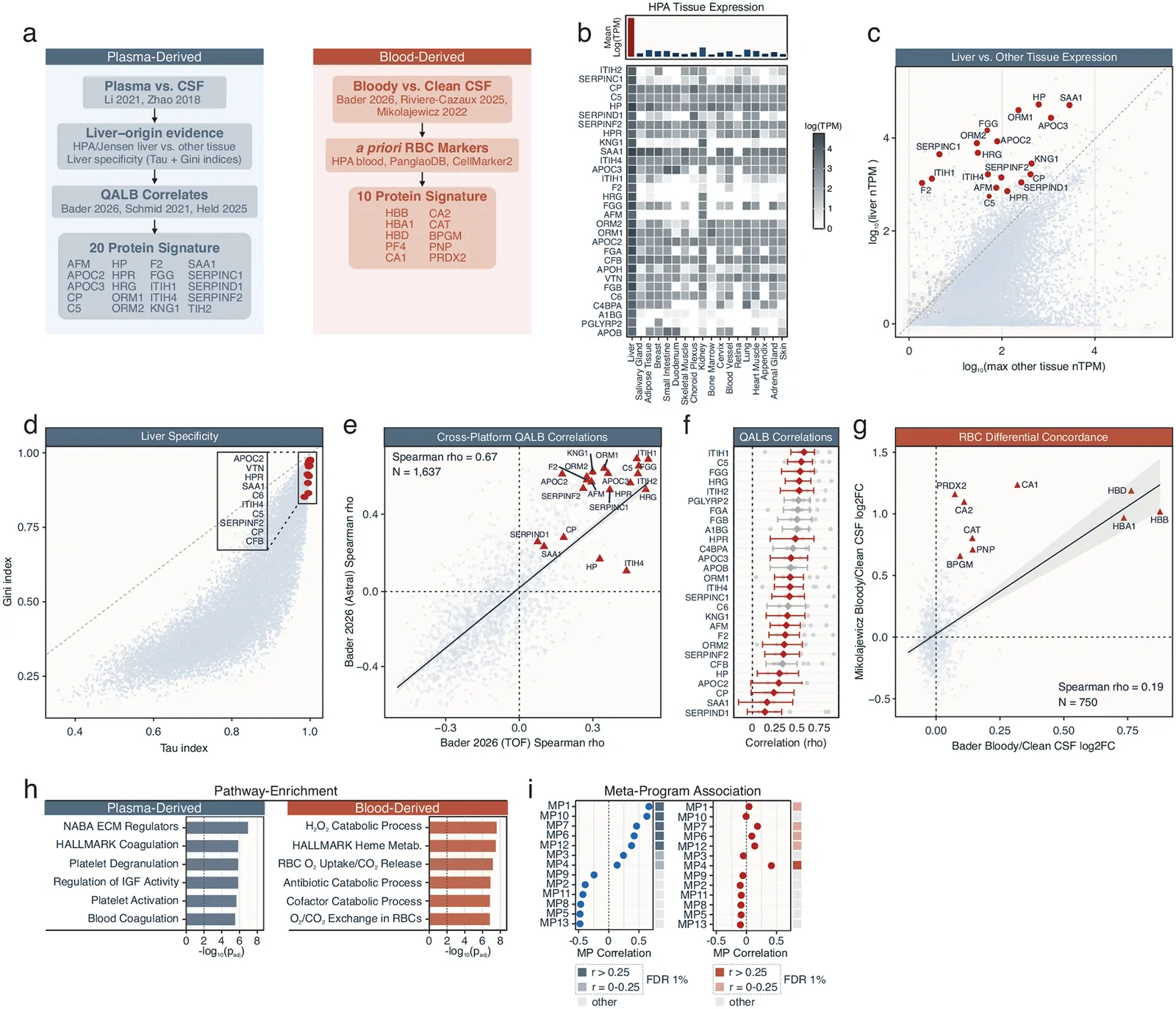
Plasma ingress and frank blood define distinct peripheral CSF signatures. **(a)** Derivation of a 20-protein plasma-ingress signature from plasma enrichment, liver specificity and association with the CSF-to-serum albumin quotient (QAlb), and a 10-protein frank-blood signature from bloody-versus-clean CSF contrasts and erythrocyte markers. **(b)** Human Protein Atlas (HPA) tissue-consensus RNA expression of plasma candidates across 36 tissues. *Colour*, log_10_-transformed nTPM; *marginal bars*, mean expression. **(c)** Liver expression versus the highest non-liver tissue. *Red triangles*, selected plasma-ingress markers. **(d)** Concordance of liver-specificity τ and Gini indices across HPA proteins (Spearman ρ = 0.93). *Inset*, most liver-specific protein. **(e)** Cross-platform concordance of protein–QAlb correlations in Bader 2026 (timsTOF versus Astral; 1,637 shared proteins; Spearman ρ = 0.67). *Red triangles*, selected markers. **(f)** Random-effects meta-analysis of protein–QAlb correlations across Bader 2026, Schmid 2021 and Held 2024. *Points*, pooled Spearman ρ; *bars*, 95% CI. **(g)** Concordance of frank-blood differential abundance between pooled Bader 2026 erythrocyte-positive versus erythrocyte-negative CSF and Mikolajewicz 2022 bloody versus clean CSF (750 shared proteins; Spearman ρ = 0.19). *Red triangles*, selected markers; *line and shading*, linear fit and 95% CI. **(h)** Top pathway enrichments for each signature. *Bars*, −log_10_ FDR from hypergeometric over-representation analysis. **(i)** Random-effects-pooled within-study correlations of plasma-ingress (*blue*) and frank-blood (*red*) scores with the 13 CSF MPs. *Points and bars*, pooled Spearman ρ and 95% CI.

**Extended Data Fig. 8.**
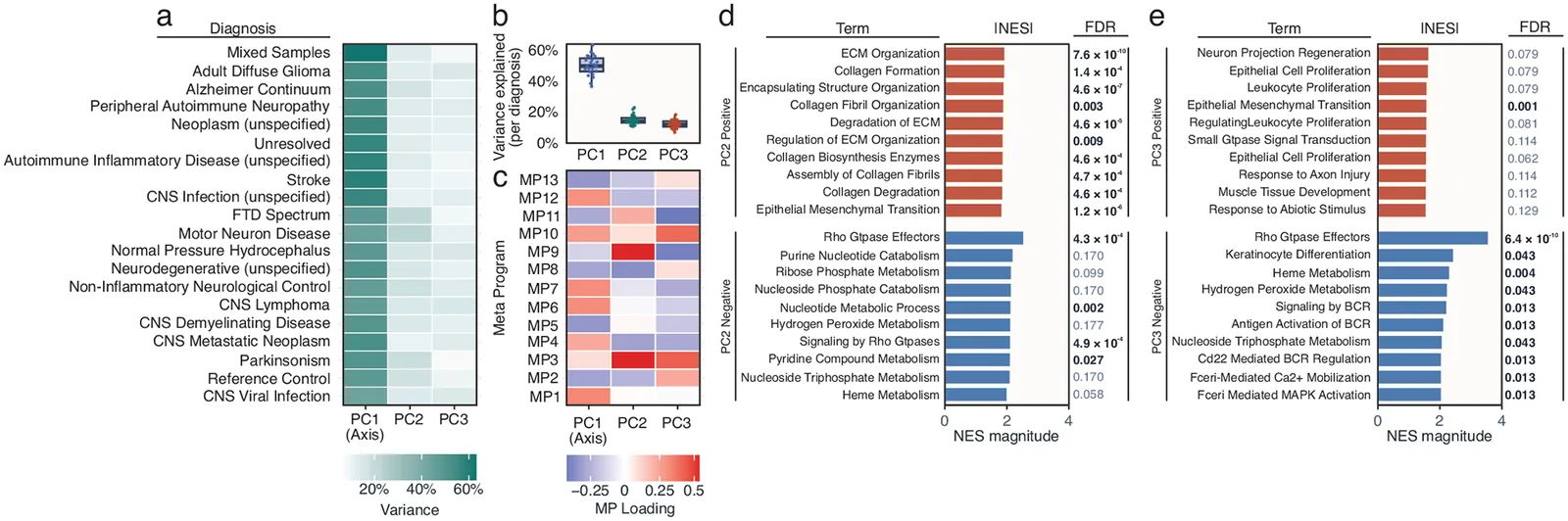
Secondary principal components capture diagnosis-spanning meta-program variation. **(a)** Variance explained by each principal-component (PC) across diagnostic groups. Fill color denotes PC variance divided by total variance (× 100). **(b)** Variance explained by each PC at the diagnostic group level. Boxes show the median and IQR. **(c)** MP loading across principal components, with positive and negative coefficients shown in red and blue, respectively. **(d, e)** Top-ranked positive- and negative-pole enrichment terms for PC2 (d) and PC3 (e), respectively. *Bar plot*, absolute normalized enrichment score (|NES|); red and blue indicate positive and negative NES, respectively. Right-hand values are Benjamini–Hochberg false discovery rates (FDR); bolded labels denote FDR < 0.05.

**Extended Data Fig. 9.**
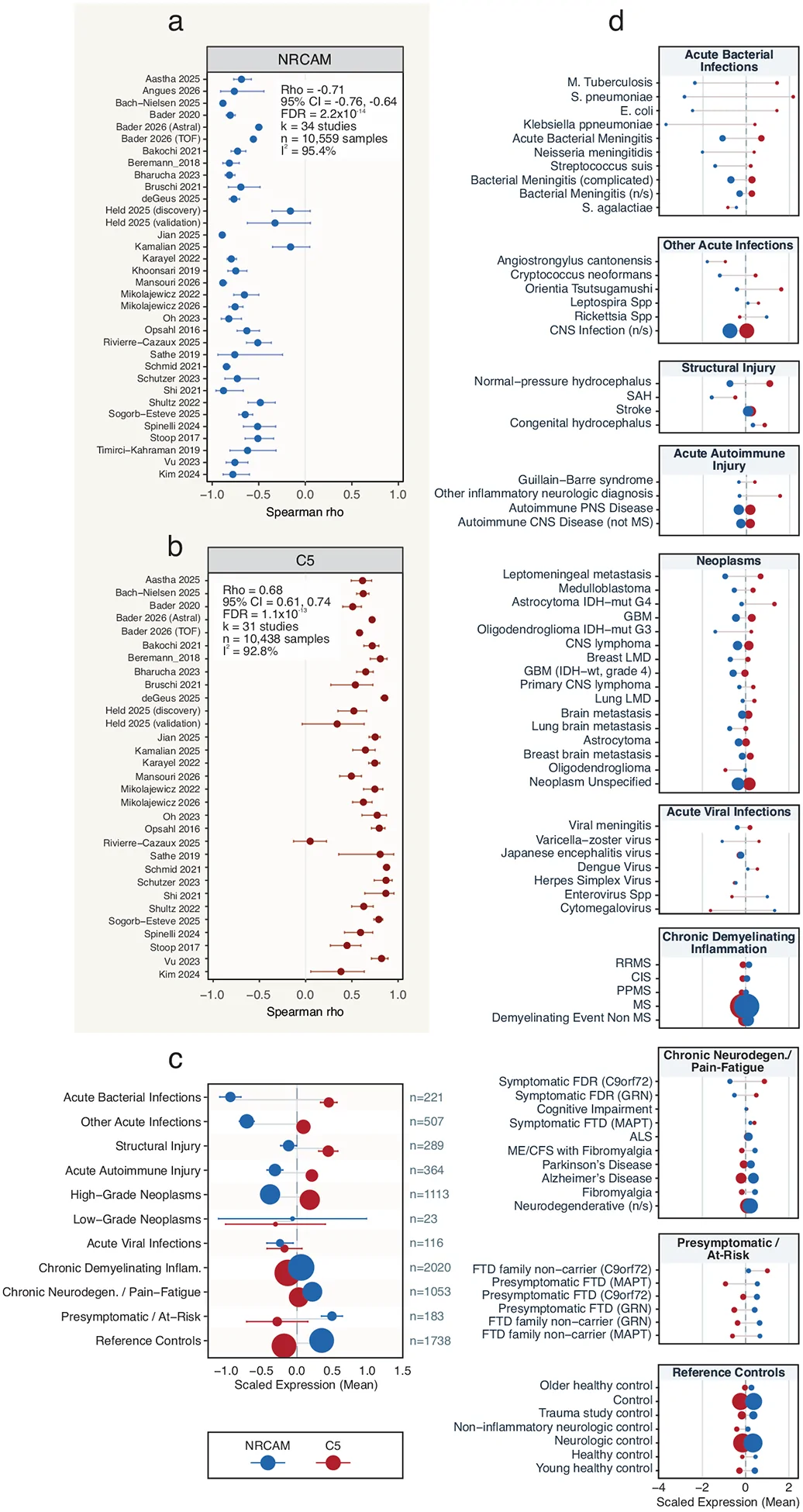
C5 and NRCAM mark opposing poles of the injury–homeostatic CSF axis across cohorts and diagnoses. **(a, b)** Study-level associations between NRCAM (**a**) or C5 (**b**) abundance and injury-homeostatic axis score. Spearman correlations were estimated within each cohort using sample-level abundance and Axis scores, then pooled using Fisher’s z random-effects meta-analysis. *Inset* shows Rho, 95% CI, FDR, k (studies), n (samples), and I^2^. **(c)** NRCAM (*blue*) and C5 (*red*) expression across diagnostic groups. Data are mean standardized expression ± 95% CI for each protein; point size is proportionate to sample size, and connecting segments show the within-diagnosis NRCAM–C5 differences. **(d)** Diagnosis-level summaries of NRCAM and C5 abundance.

**Extended Data Fig. 10.**
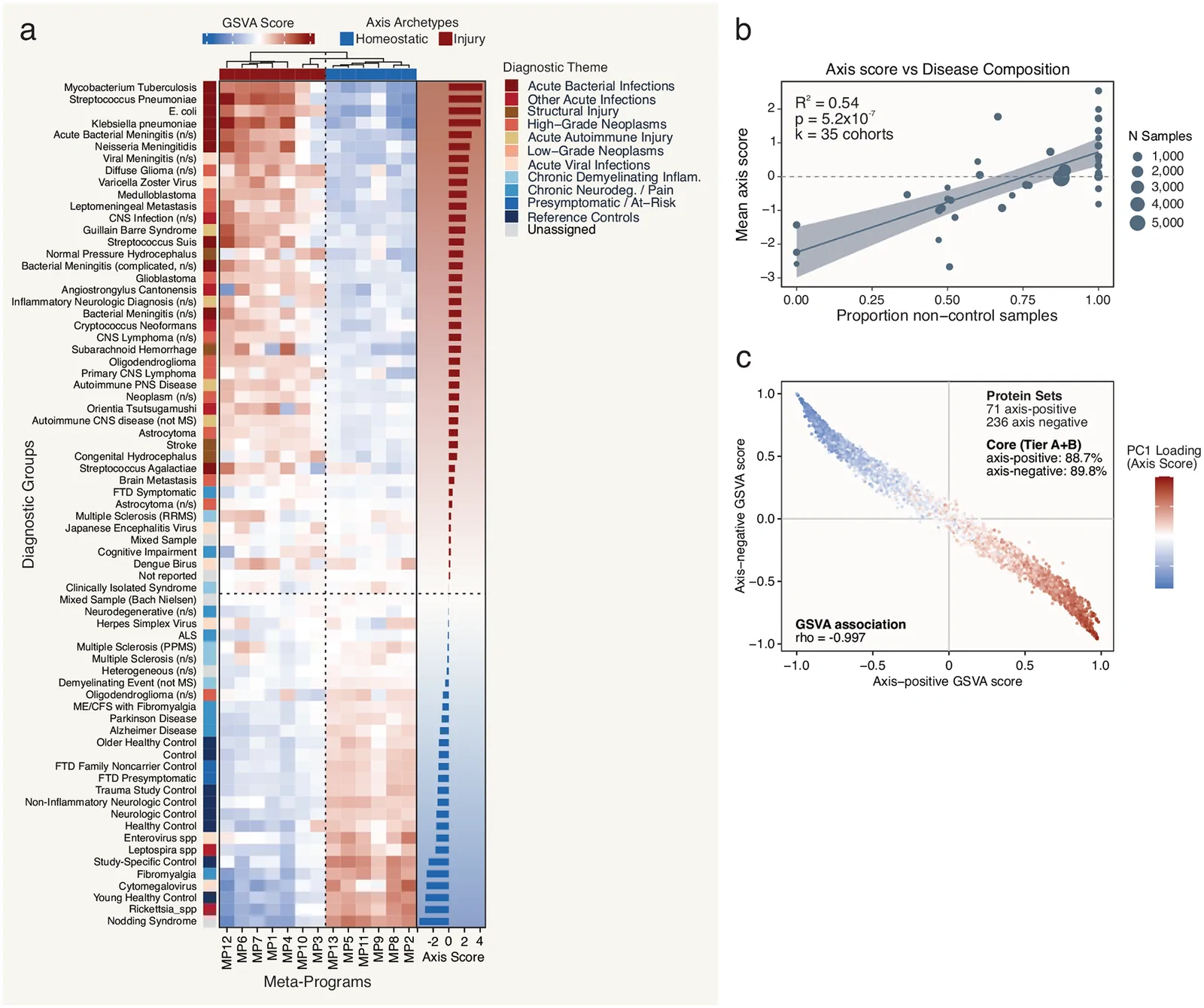
The injury–homeostatic meta-program axis organizes CSF proteomic states across diagnoses. **(a)** Heatmap of mean GSVA activity for the 13 consensus CSF MPs across diagnoses from 35 cohorts. *Rows*, diagnosis groups rank-ordered by mean Axis score; *columns*, MPs rank-ordered by PC1 loading. **(b)** Sample-level association between cohort composition and mean Axis score. *X-axis*, proportion of non-control samples; *point size*, sample size; *line*, linear OLS fit with 95% CI. **(c)** Protein-set surrogate reconstruction of the axis using GSVA. Axis-positive and axis-negative protein sets were defined from the pooled protein–axis association (|ρ| ≥ 0.40 and FDR ≤ 0.05; **Supplementary Tables 4 and 13**). Each point represents one sample, plotted by axis-positive and axis-negative GSVA scores and coloured by the corresponding PC1-derived axis score. GSVA, gene set variation analysis; PC1, principal component 1

**Extended Data Fig. 11.**
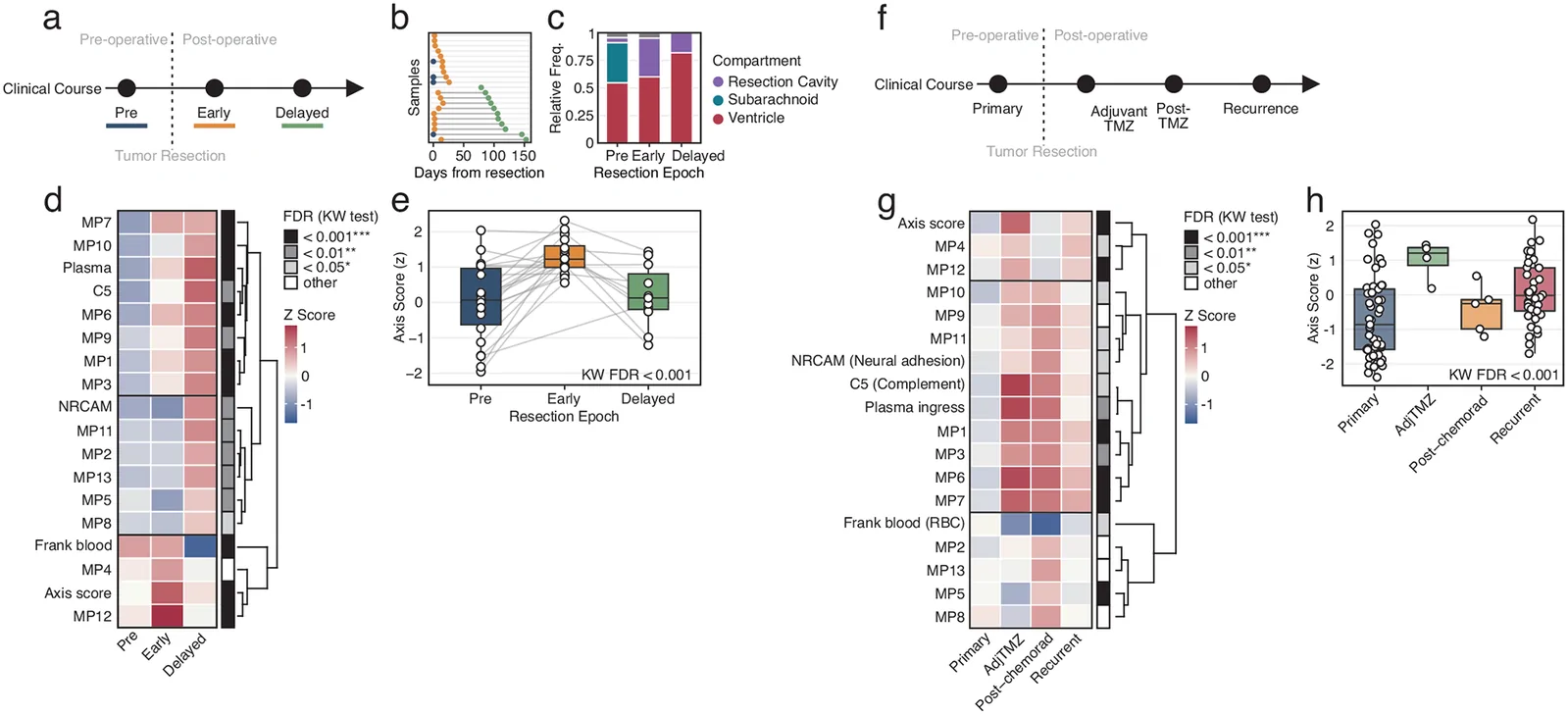
Resection and treatment shape glioma CSF injury, leakage and meta-program activity. **(a)** Schematic of the resection-anchored longitudinal design; samples grouped as pre-operative (Pre, n = 22), early post-operative (Early, n = 20) and delayed post-operative (Delayed, n = 11) relative to tumour resection. **(b)** Overview of samples with respect to days from resection. **(c)** Relative frequency of CSF sampling compartment (resection cavity, subarachnoid, ventricle) across resection epochs. **(d)** Mean z scores across resection epochs for meta-programs (MP1–MP13), the injury–homeostasis axis score, C5 and NRCAM markers, and plasma-ingress and frank-blood signatures. Rows are hierarchically clustered (Euclidean distances); side bar shows FDR for the epoch effect per feature. **(e)** Axis-score (z) distributions across resection epochs. Boxes show median and interquartile range with whiskers to 1.5× IQR; points are individual samples and lines connect paired samples. **(f)** Schematic of the treatment-state analysis spanning primary, adjuvant temozolomide (TMZ), post-chemoradiation and recurrent disease. **(g)** Treatment-state map of the same 18 features using therapy-state–standardized z scores; row clustering and FDR side bar as in (d). **(h)** Axis-score (z) distributions across the four treatment states (primary, n = 53; adjuvant TMZ, n = 4; post-chemoradiation, n = 5; recurrent, n = 37); box plot as in (e).

**Extended Data Fig. 12.**
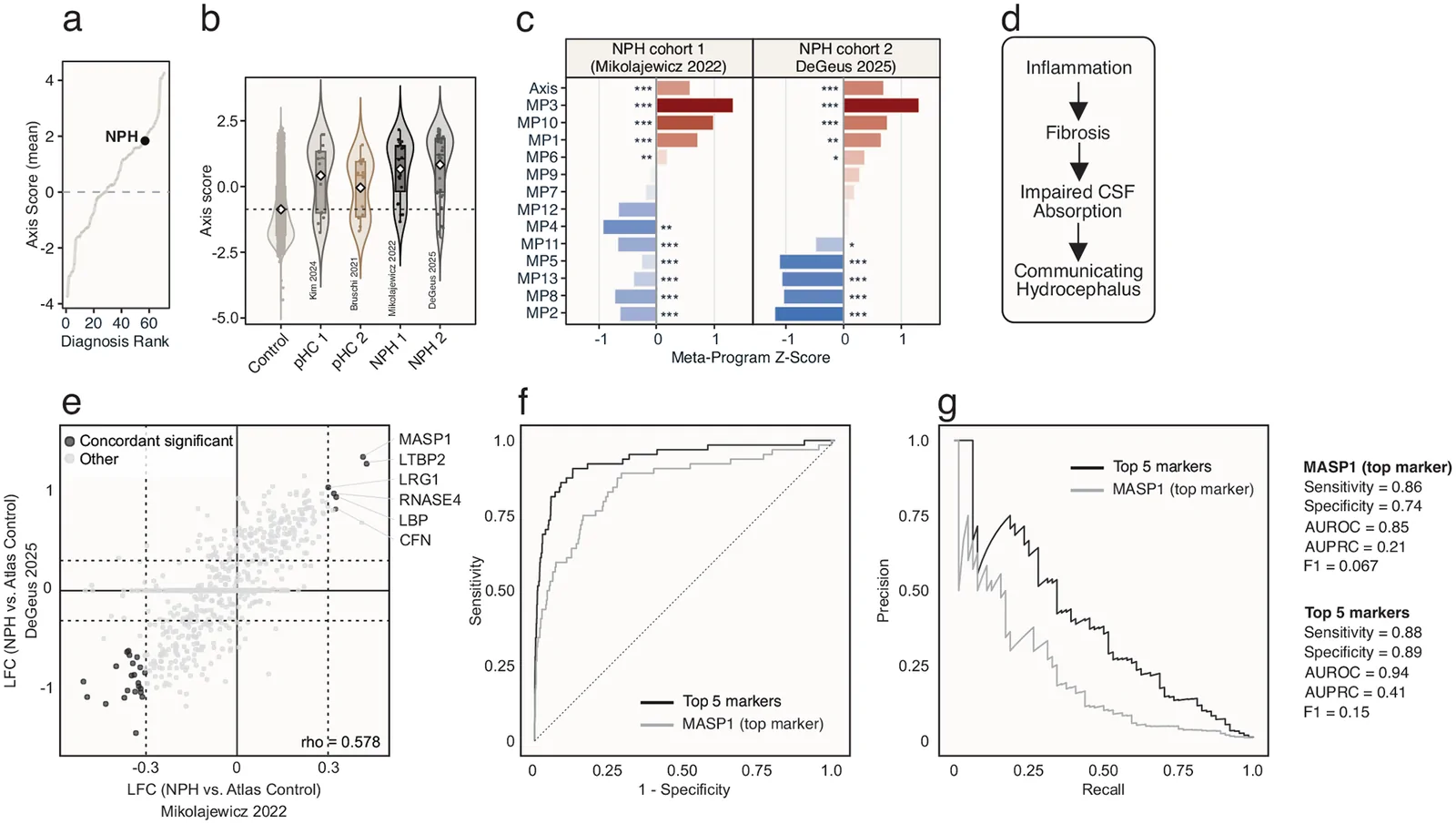
NPH is characterized by convergent inflammatory-fibrotic CSF signatures. **(a)** CSF injury–homeostatic axis score across atlas diagnoses, ranked low to high; NPH position along the spectrum is indicated. **(b)** Sample-level axis-score distributions for atlas controls, two pediatric hydrocephalus cohorts (pHC1, Kim 2024; pHC2, Bruschi 2021), and two adult NPH cohorts (Mikolajewicz 2022, n = 20; de Geus 2025, n = 44). Points, individual samples; violins and box plots, group distributions; diamonds, means; dashed line, axis zero. **(c)** Axis and meta-program (MP) activity in the two adult NPH cohorts. Bars show z-scores relative to controls; red, enrichment; blue, depletion. BH-adjusted Wilcoxon p values, *FDR<0.05, **FDR<0.01, ***FDR<0.001. **(d)** Proposed NPH model: inflammatory activation drives fibrotic remodeling, impairing CSF absorption and producing communicating hydrocephalus. **(e)** Cross-cohort comparison of NPH-associated protein log fold changes (Mikolajewicz 2022 vs. de Geus 2025) among 917 shared proteins. Dashed lines, |logFC| = 0.30; dark points, proteins with concordant significance (FDR ≤ 0.05; n = 28); grey points, all other proteins. **(f, g)** Receiver operating characteristic (f) and precision–recall (g) curves from stratified five-fold cross-validated weighted logistic regression distinguishing pooled adult NPH from all other atlas diagnoses. MASP1 alone: AUROC = 0.85, AUPRC = 0.21. Five-marker panel (MASP1, LTBP2, RNASE4, ENO2, L1CAM): AUROC = 0.94, AUPRC = 0.41

## Supplementary Material

1

**Supplementary Table 1.** Overview of the 35 cerebrospinal fluid (CSF) proteomic datasets (2016–2026) assembled in this study, spanning neuro-oncology, neurodegenerative, neuroinflammatory, infectious, paediatric, and control cohorts

**Supplementary Table 2.** Diagnosis dictionary.

**Supplementary Table 3.** Meta-analysis of MS-assayable protein detection in cerebrospinal fluid.

**Supplementary Table 4.** Protein lists derived from the CSF meta-atlas.

**Supplementary Table 5.** Observed and model-predicted detection rates for 2,762 proteins.

**Supplementary Table 6.** Core CSF proteome features used to annotate the latent-class model that defines the six Core Classes (CC1–CC6).

**Supplementary Table 7.** Benchmark of batch-correction methods for proteomics.

**Supplementary Table 8.** Catalog of 247 latent component programs and their assignments to 13 consensus meta-programs.

**Supplementary Table 9.** Meta-program LOSO transferability and variance-explained analyses.

**Supplementary Table 10.** Functional enrichment for the 13 consensus CSF proteomic meta-programs.

**Supplementary Table 11.** Single-cell projection of meta-program (MP) and axis scores onto reference cell-type atlases.

**Supplementary Table 12.** Plasma-ingress and frank-blood signatures.

**Supplementary Table 13.** Meta-program protein correlations and consensus axis signature.

**Supplementary Table 14.** Cross-disease clinical associations of CSF proteomic features.

**Supplementary Table 15.** CSF proteomics and classifier development for normal pressure hydrocephalus (NPH).

**Supplementary Table 16.** C5–NRCAM and injury–homeostasis axis prediction models.

This is a list of supplementary files associated with this preprint. Click to download.

- SupplementalTable4ProteinlistsfromCSFmetaatlas.xlsx
- SupplementalTable8Catalogof247latentprograms13metaprograms.xlsx
- SupplementalTable12Plasmaingressandfrankbloodsignatures.xlsx
- SupplementalTable14Crossdiseaseclinicalassociations.xlsx
- SupplementalTable15CSFproteomicsNPHclassifier.xlsx
- SupplementalTable2Diagnosisdictionary.xlsx
- SupplementalTable7Benchmarkofbatchcorrectionmethods.xlsx
- SupplementalTable9MetaprogramLOSOtransferability.xlsx
- SupplementalTable1Overviewof35CSFdatasets.xlsx
- SupplementalTable5Observedvs.predicteddetectionrates.xlsx
- SupplementalTable16C5XXXNRCAMaxispredictionmodels.xlsx
- SupplementalTable3MSassayableproteindetectionmetaanalysis.xlsx
- SupplementalTable11SinglecellprojectionofMPaxisscores.xlsx
- SupplementalTable10Functionalenrichmentfor13metaprograms.xlsx
- SupplementalTable6CoreproteomefeaturesCC1XXXCC6.xlsx
- SupplementalTable13Metaprogramproteincorrelationsaxissignature.xlsx

## Figures and Tables

**Figure 1.**
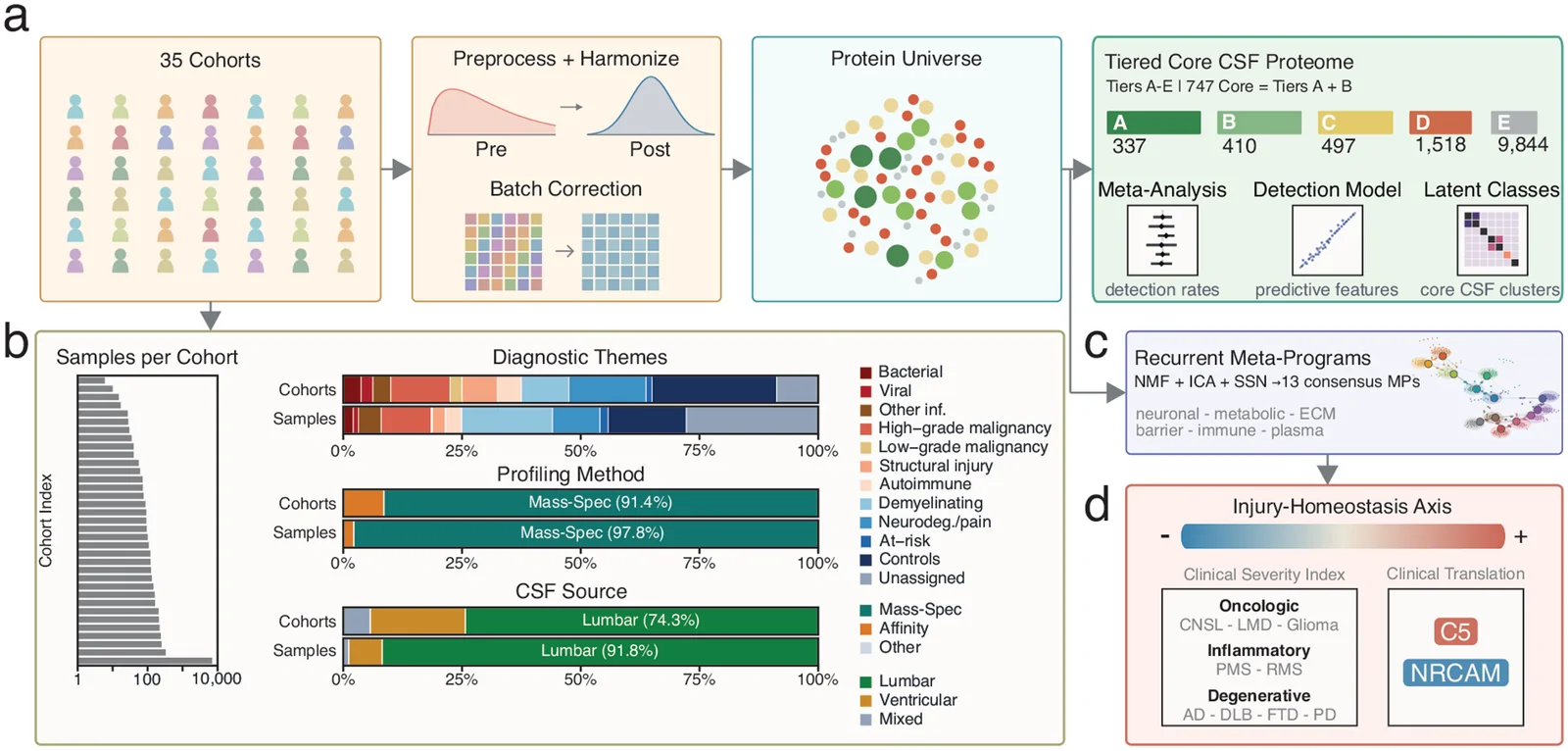
Construction of a cross-cohort CSF proteomic meta-atlas and derivation of an injury–homeostasis axis. **(a)** Atlas construction workflow. Proteomic profiles from 35 CSF cohorts were preprocessed within study, and shared matrices were harmonized for atlas-wide analyses. Coverage-aware meta-analysis of protein detection across 21 discovery mass-spectrometry studies stratified 12,606 proteins into five evidence tiers: A, 337; B, 410; C, 497; D, 1,518; and E, 9,844. Tiers A and B define the 747-protein core mass-spectrometry-accessible CSF proteome. Detection modelling and latent-class analysis characterized the determinants and biological organization of recurrently detected proteins. **(b)** Composition of the quality-controlled atlas comprising 10,560 samples. Cohorts are ordered by sample size on a logarithmic scale (*left*); stacked bars show cohort- and sample-level distributions by diagnostic theme, proteomic profiling method and CSF sampling site (*right*). **(c)** Recurrent CSF proteomic meta-programs. Independent within-cohort decomposition by non-negative matrix factorization (NMF), independent component analysis (ICA) and scale-free shared-nearest-neighbour network analysis (SSN) yielded 13 consensus meta-programs spanning neuronal–synaptic, metabolic, extracellular-matrix, barrier–epithelial, immune–inflammatory and plasma–complement biology. **(d)** Clinical organization and translation of the dominant axis. The first principal component of meta-program activity defines a continuous injury–homeostasis gradient, extending from neuronal–synaptic homeostasis (negative, *blue*) to complement-, plasma- and inflammation-associated injury (positive, *red*). Axis activity tracked adverse clinical features in neuro-oncological cohorts, distinguished progressive from relapsing–remitting multiple sclerosis and was enriched across four neurodegenerative diagnoses. Complement component 5 (C5) and neuronal cell adhesion molecule (NRCAM) mark the opposing poles and provide a candidate two-protein approximation of the axis. CNSL, central nervous system lymphoma; LMD, leptomeningeal disease; MS, multiple sclerosis; AD, Alzheimer’s disease; DLB, dementia with Lewy bodies; FTD, frontotemporal dementia; PD, Parkinson’s disease.

**Figure 2.**
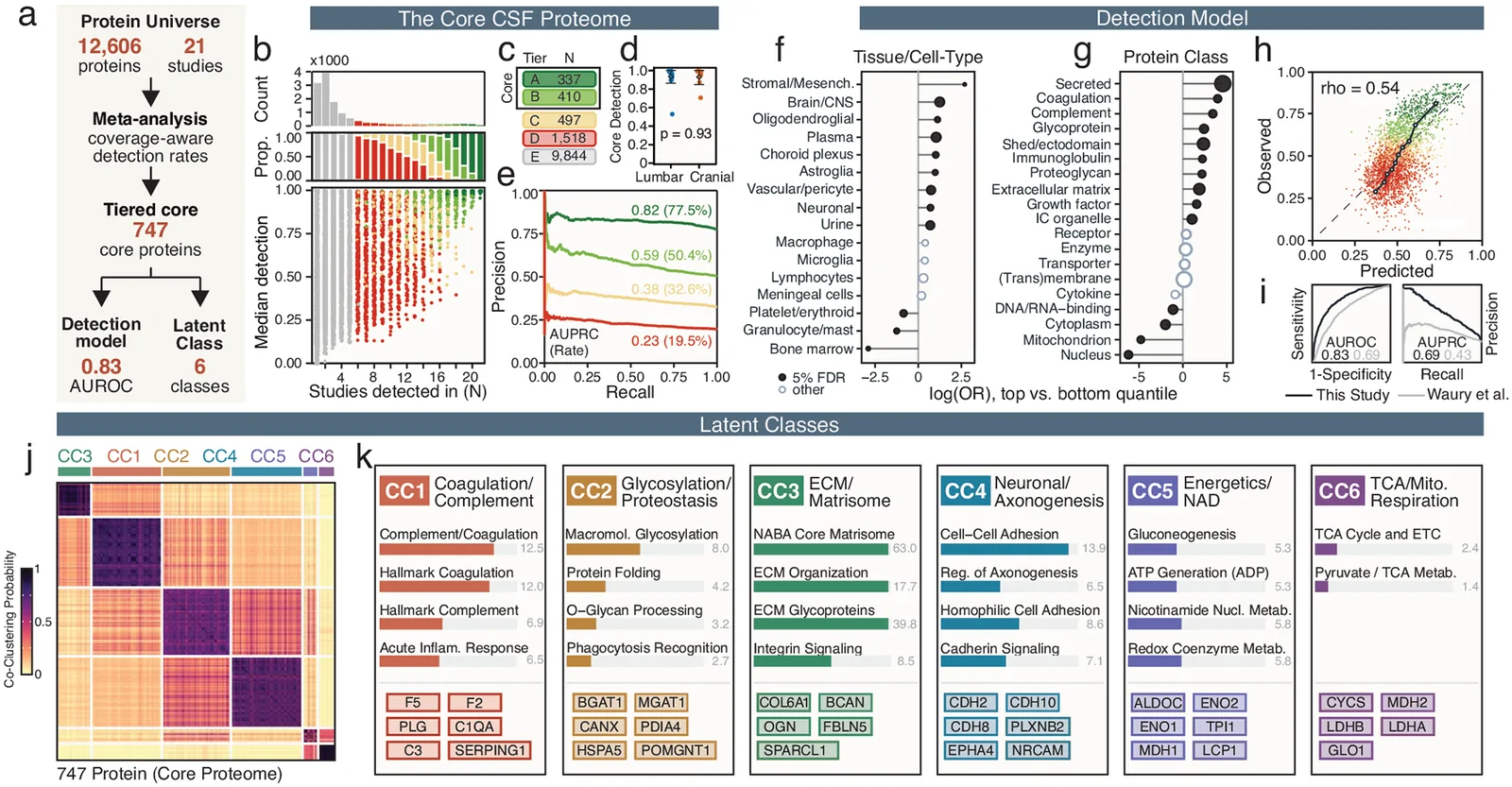
The mass-spectrometry-accessible core CSF proteome and its biological organization. **(a)** Workflow for defining the core CSF proteome. Detection tiers assigned by coverage-aware detection (CAD) lower bound 95% CI: Tier A, ≥0.80; Tier B, ≥0.50 to <0.80; Tier C, ≥0.20 to <0.50; Tier D, <0.20; Tier E, insufficient evidence (Supplementary Note 1). **(b)** Protein detection by number of studies. Absolute (*upper*) and proportional (*middle*) protein counts; *M*edian within-study detection (*bottom*). Colored by detection tier. **(c)** Number of proteins per detection tier. **(d)** Detection rates in core CSF proteome (Tier A/B) in lumbar versus cranial CSF, compared across studies (n = 14 lumbar, 8 cranial). **(e)** External validation in 12 hold-out CSF studies. Precision–recall curves show recovery of tiered proteins; AUPRC and observed held-out detection rate are indicated. **(f, g)** Biological associations of CSF detectability. Data are log_2_ odds ratios comparing proteins in the highest (n = 553) versus lowest (n = 553) quintiles of observed detection for tissue/cell (f) or protein-class (g) annotations. **(h, i)** CSF protein detection model performance. Predicted detection rate correlated with observed detection across proteins (h; *dashed line*, identity; *solid line*, decile means) and discriminated Tier A/B core proteins from non-core candidates (i; AUROC = 0.83, AUPRC = 0.69), outperforming the Waury et al. CSF model on the same benchmark^14^. **(j)** Consensus co-clustering matrix used to resolve six core classes (CC1-CC6). Protein class probabilities were estimated by latent-class modeling using tissue origin, subcellular compartment, and release mechanism features. *Fill colour*, pairwise co-cluster probability; *annotated margin*, the six core classes (CC1–CC6). **(k)** Functional annotation of the six core classes. *Bars*, top enriched pathways as −log10(FDR); *boxed labels*, representative markers.

**Figure 3.**
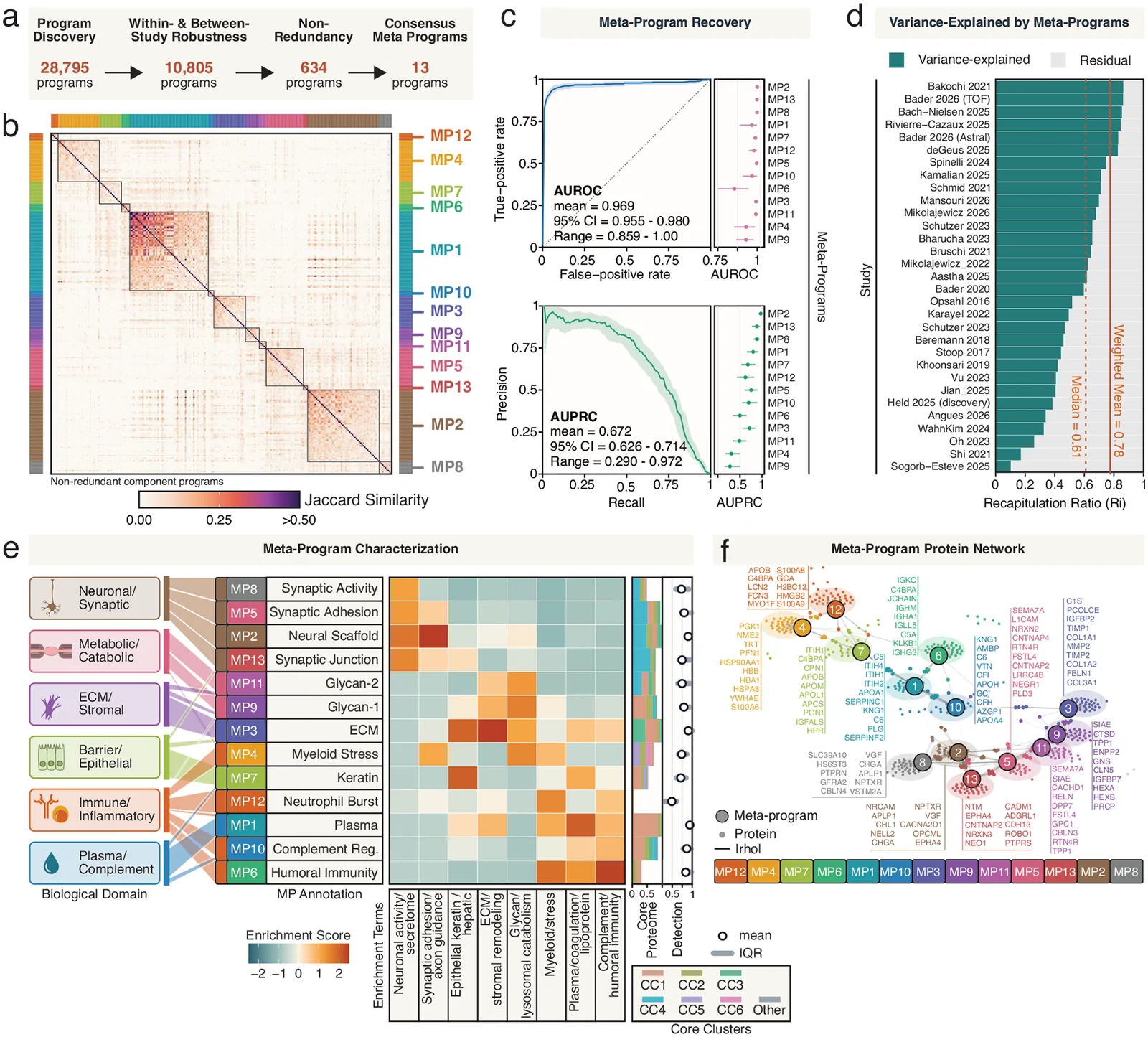
Derivation, validation, and biological characterization of 13 consensus CSF proteomic meta-programs. **(a)** Meta-program discovery workflow. Within-cohort non-negative matrix factorization (NMF) and independent component analysis (ICA) across ranks *K*=3–15, together with scale-free shared-nearest-neighbour (SSN) analysis across multiple resolutions, generated 28,795 candidate programs. Sequential filtering retained 10,805 robust and 634 non-redundant programs, of which 247 were assigned by iterative community detection to 13 consensus meta-programs (MP1–MP13). **(b)** Pairwise Jaccard similarity among the 247 assigned programs, ordered by hierarchical clustering. *Marginal bars*, consensus meta-program. (**c)** Leave-one-study-out recovery across 35 held-out datasets. Receiver operating characteristic and precision–recall curves compare correlations between predicted and observed MP activity with sample-size-matched Gaussian null correlations. *Right*, MP-specific AUROC and AUPRC; *points and bars*, estimates ± 95% CI. **(d)** Study-level ceiling-normalized recapitulation ratio (*R*_*i*_) in 33 evaluable cohorts, calculated as the cross-validated fraction of variance explained (CV-FVE) by the MPs relative to a PCA ceiling using the number of active MPs in each study. *Teal*, recapitulated variance; *grey*, residual; *dashed and solid lines*, median and sample-size-weighted mean *R*_*i*_, respectively. **(e)** Integrated biological characterization. Alluvial links assign MPs to six biological domains; the heatmap shows scaled enrichment scores for representative annotations, and right-hand annotations summarize core-proteome composition and detection properties. **(f)** Bipartite network linking the 13 MPs to member proteins. *Protein-node size*, number of MP memberships; *Grey edges*, membership; *edge width*, proportional to the protein–MP Spearman correlation; *coloured labels*, ten most positively correlated proteins for each MP.

**Figure 4.**
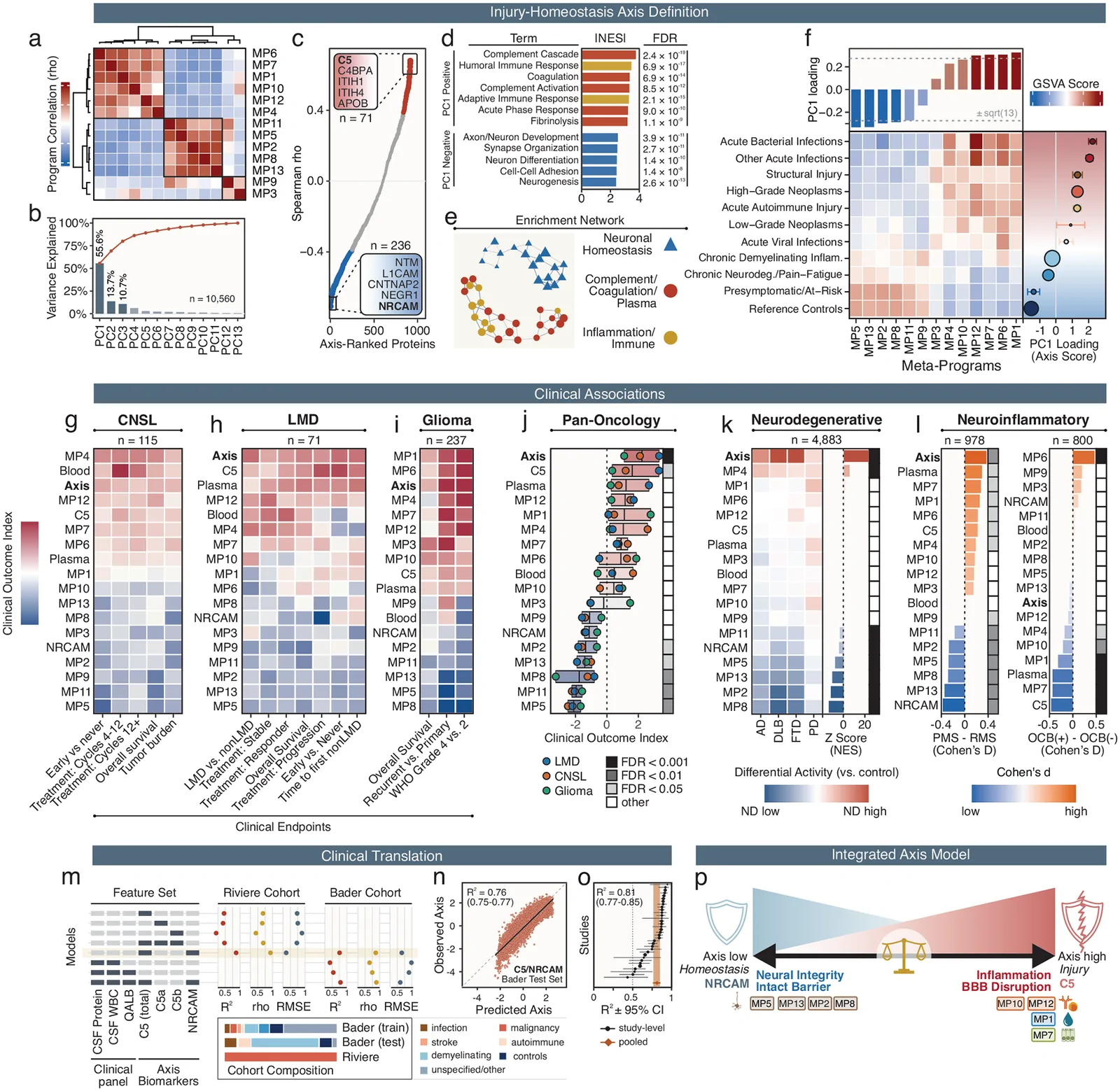
A CSF injury–homeostasis axis tracks disease severity and can be approximated by C5 and NRCAM. **(a)** Hierarchical clustering of random-effects-pooled pairwise correlations among the 13 CSF meta-program (MP) activities. **(b)** PCA of GSVA-derived MP activities across 10,560 CSF samples. *Bars*, variance explained; *line*, cumulative variance. Significance assessed against 1,000 permutations (*dark bars*, FDR < 0.05; *muted bars*). **(c)** Proteins ranked by random-effects-pooled Spearman correlation with PC1. Axis-positive (red; *n* = 71) and axis-negative (blue; *n* = 236) proteins were defined by |ρ| ≥ 0.40 and FDR ≤ 0.05. **(d)** GSEA of proteins ordered by pooled PC1 association. *Bars*, absolute normalized enrichment score (|NES|). **(e)** Network of significantly enriched gene sets associated with PC1. *Nodes*, gene sets; edges, Jaccard overlap. **(f)** Diagnostic groups ranked by mean injury–homeostasis axis score. *Heatmap*, mean GSVA-derived MP activity; *upper margin*, MP loadings on PC1; *right margin*, mean axis scores with 95% CI. (**g–i)** Multiple clinical endpoint analysis of the axis and MPs in central nervous system lymphoma (CNSL; **g**), leptomeningeal disease (LMD; **h**) and glioma (**i**). CNSL endpoints comprised early versus absent treatment response (*n* = 34), treatment stage (*n* = 115), overall survival (OS; *n* = 33) and tumour burden (*n* = 30). LMD endpoints comprised LMD status (*n* = 71), treatment response (*n* = 90), OS (*n* = 23), early versus absent treatment response (*n* = 23) and time to first LMD-negative assessment after treatment (*n* = 16). Glioma endpoints comprised OS (*n* = 137), primary versus recurrent disease (*n* = 115) and WHO grade 2 versus grade 4 disease (*n* = 237). Binary, continuous and time-to-event endpoints were analysed using logistic regression, correlation or linear models, and Cox regression, respectively. *Red*, associations with adverse states or outcomes; *blue*, favourable associations. **(j)** Pan-oncology synthesis of clinical associations. *Points*, pooled endpoints (per diagnosis); *boxes*, median and IQR, *marginal tiles*; significance (*shaded*, FDR < 0.05). **(k)** Differential axis and MP profiles in an external neurodegenerative cohort comprising Alzheimer’s disease (AD; *n* = 1,883), dementia with Lewy bodies (DLB; *n* = 763), frontotemporal dementia (FTD; *n* = 772) and Parkinson’s disease (PD; *n* = 1,465)^82^. **(l)** Axis and MP differences between progressive and relapsing–remitting multiple sclerosis (*n* = 978), and between oligoclonal-band-positive and -negative CSF (*n* = 800). *Bars*, standardized mean differences (Cohen’s *d*). **(m)** Linear models approximating the injury–homeostasis axis. Models in the Riviere-Cazaux cohort compare intact C5, C5a, C5b–6 and NRCAM combinations; models in the Bader cohort compare C5 and NRCAM with total CSF protein, leukocyte count and the CSF-to-serum albumin quotient (QAlb). The target was injury-homeostasis score calculated as the eigenscore of GSVA enrichment for axis-positive and axis-negative protein sets, with C5 and NRCAM removed before scoring to prevent target leakage. Bader models were trained in the Astral subset (*n* = 1,627) and evaluated without refitting in held-out pooled timsTOF subsets (*n* = 4,854). Performance was assessed by R^2^, Spearman ρ and root-mean-square error (RMSE). **(n)** Observed versus C5–NRCAM-predicted axis scores in held-out Bader timsTOF samples. *Dashed line*, identity; *solid line*, linear fit. **(o)** Random-effects meta-analysis of cohort-specific R^2^ values for C5–NRCAM models across 34 cohorts and 10,559 samples. **(p)** Integrated model contrasting low-axis neuronal–synaptic homeostasis and CNS barrier integrity with high-axis complement- and plasma-associated inflammation, barrier disruption and injury.

## Abbreviations

AD: Alzheimer’s disease
ASW: average silhouette width
AUPRC: area under the precision–recall curve
AUROC: area under the receiver operating characteristic curve
BERT: Batch-Effect-Reduction-Tree
BH: Benjamini–Hochberg
CAD: coverage-aware detection
CI: confidence interval
CND: coverage-naive detection
CNS: central nervous system
CNSL: central nervous system lymphoma
CC1–CC6: Core Classes 1–6 (of the core CSF proteome)
CSF: cerebrospinal fluid
CV-FVE: cross-validated fraction of variance explained
DDA: data-dependent acquisition
DIA: data-independent acquisition
DLB: dementia with Lewy bodies
ECM: extracellular matrix
EVD: external ventricular drain
FC: fold change
FDR: false discovery rate
FTD: frontotemporal dementia
FVE: fraction of variance explained
GEO: Gene Expression Omnibus
GO: Gene Ontology
GSEA: gene-set enrichment analysis
GSVA: gene-set variation analysis
HPA: Human Protein Atlas
HVP: highly variable protein
ICA: independent component analysis
IQR: interquartile range
kNN: k-nearest neighbours
KEGG: Kyoto Encyclopedia of Genes and Genomes
LC-MS/MS: liquid chromatography–tandem mass spectrometry
LMD: leptomeningeal disease
LOSO: leave-one-study-out
LP: lumbar puncture
MP: meta-program
MS: mass spectrometry
MSE: mean squared error
NAD: nicotinamide adenine dinucleotide
ND: neurodegenerative
NES: normalized enrichment score
NfL: neurofilament light
OLS: ordinary least squares
NMF: non-negative matrix factorization
NPH: normal-pressure hydrocephalus
OCB: oligoclonal band
ORA: over-representation analysis
OS: overall survival
PC1: first principal component
PCA: principal component analysis
PCR: principal component regression
PD: Parkinson’s disease
PEA: proximity extension assay
QAlb: CSF/serum albumin quotient
REML: restricted maximum likelihood
RMSE: root-mean-square error
ROC: receiver operating characteristic
SILAC: stable isotope labelling by amino acids in cell culture
SMD: standardized mean difference
SSN: scale-free shared-nearest-neighbour network
TAMPOR: tunable adjustment of median polish of ratio
TCA: tricarboxylic acid
TMT: tandem mass tag
TMZ: temozolomide
TPM: transcripts per million
UMAP: uniform manifold approximation and projection
WHO: World Health Organization
