# Supplementary material for: A shared injury–homeostasis gradient in the human CSF proteome across neurological disease
Source: Res Sq. 2026 Aug 11:rs.3.rs-10480838. Preprint. [Version 1] doi: 10.21203/rs.3.rs-10480838/v1 (PMC13640887; doi:10.21203/rs.3.rs-10480838/v1)
Supplement: Supplementary file 1 [file NIHPPRS10480838V1-supplement-1.pdf]

8 Supplementary Notes

8.1 Supplementary Note 1. Coverage-aware detection meta-analysis of the core CSF proteome

Defining a recurrent CSF proteome across heterogeneous mass-spectrometry studies requires distinguishing true biological absence from technical non-observation. We therefore developed a coverage-aware detection framework that first estimated the assayable proteome for each study. The assayable proteome was defined as the subset of proteins each study could plausibly detect given its analytical depth and platform, rather than those directly observed (**Extended Data Fig. 1a**), then performed random-effects meta-analysis of detection only across informative study contexts.

8.1.1 Informative CSF mass-spectrometry datasets

We restricted this analysis to a subset of structurally informative CSF mass-spectrometry datasets, excluding studies in which we could not reliably distinguish non-assayable or non-detected proteins from true absence because of upstream filtering or incomplete reporting. Eligible datasets were required to be non-panel CSF MS studies with sample-level protein matrices, nonzero sample and protein counts, and no prefiltering. Olink affinity[40] and SomaScan aptamer[49] datasets were excluded from derivation because their observed protein universe is primarily defined by assay design rather than empirical MS detectability. Among structurally eligible MS datasets, we further required evidence that the matrix retained prevalence information, defined as mean protein missingness >2%, or interquartile range of protein missingness >5%. Dense matrices failing these thresholds were excluded from derivation because they lacked sufficient non-detection structure to support protein-level assayability inference (**Supplementary Table 3**). This yielded 21 study-level MS datasets.

8.1.2 Assayable-universe derivation

The 21 derivation studies were ordered by observed protein depth, and each study was assigned a centred rolling neighbourhood along this depth axis. For each index study, a protein was considered assayable if it was detected in the index study itself or received sufficient support from depth-matched peer studies in the local window.

We selected a three-study centred window, with a maximum of 700 added proteins and a median of 131 added proteins per study. Across the 21 derivation studies, rolling support expanded study-specific protein universes by a median of 10.3% (**Extended Data Fig. 1b**). Final universes spanned 338–11,010 proteins. Coverage-aware detection was defined as the fraction of assayable studies in which a protein was observed (**Supplementary Table 3**).

8.1.3 Coverage-aware detection meta-analysis

Proteins assayable in at least two studies were synthesized using restricted maximum-likelihood random-effects meta-analysis. Pooled detection rates and 95% confidence intervals were estimated using Hartung-Knapp-Sidik-Jonkman adjustment, providing conservative uncertainty estimates for proteins supported by small numbers of studies. Tier assignment was based on the lower bound of the pooled 95% confidence interval (**Supplementary Table 3**).

Proteins were assigned to detection tiers using fixed lower-bound thresholds: Tier A, at least 0.80; Tier B, at least 0.50 and less than 0.80; Tier C, at least 0.20 and less than 0.50; and Tier D, less than 0.20. Candidates underwent subsequent leave-one-study-out sensitivity analysis, in which the meta-analysis was refit after sequential removal of each contributing study. Proteins for which more than 20% of deleted-study fits crossed a tier boundary were reassigned by majority rule to the median deleted-study class. A final support gate then required at least six informative studies; proteins below this threshold were assigned to Tier E Insufficient Evidence regardless of their pooled estimate. Tier A and B were collectively defined as the 747-protein core CSF proteome.

8.1.4 Comparison with coverage-naive detection

We next compared coverage-aware detection (CAD) with a coverage-naive approach in which non-detection was evaluated without study-specific assayability correction. Coverage-naive detection (CND) substantially inflated strict-core calls (**Extended Data Fig. 1c**). Of the Tier A proteins, 305 were shared, whereas 32 were unique to the coverage-aware framework and 405 were unique to the coverage-naive framework (**Extended Data Fig. 1d**). This indicates that denominator choice materially affects core-proteome inference.

8.1.5 Orthogonal validation

Coverage-aware detection was benchmarked against the high-confidence CSF reference from Waury et al. using the pooled lower 95% detection bound as the ranking score[14]. Coverage-aware detection outperformed coverage-naive detection in precision-recall analysis, with AUPRC 0.25 (95% CI 0.23–0.27) versus 0.22 [95% CI 0.20–0.24; delta = 0.030 (95% CI 0.019–0.040, $P = 5.0 \times 10^{-3}$); **Extended Data Fig. 1e**] and in ROC analysis, with AUROC 0.68 (0.66–0.70) versus 0.56 [95% CI 0.54–0.58; delta = 0.12 (0.10–0.13), $P = 5.0 \times 10^{-3}$; **Extended Data Fig. 1f**]. This gain indicates improved global rank separation of high-confidence CSF proteins after assayability correction.

8.2 Supplementary Note 2. Benchmarking matrix-correction strategies for the CSF meta-atlas

We benchmarked whether matrix-correction methods could remove batch structure while preserving biological structure (**Extended Data Fig. 4, Supplementary Table 7**). We evaluated

nine matrix-correction methods against the uncorrected reference, yielding ten matrix states: uncorrected, limma, ComBat, quantile normalization, TAMPOR, WaveICA, Scanorama, BERT-limma, BERT-ComBat and BERT-TAMPOR. Benchmarking was performed on a shared evaluation matrix of 917 proteins comprising the core CSF proteome and meta-program proteins across 10,560 samples. We also included BERT-based correction variants to evaluate a sparser expanded matrix of 3,000 proteins.

Performance was assessed using nine direction-aware metrics grouped into batch-removal and biology-preservation domains (**Extended Data Fig. 4a,b**). The batch-removal domain comprised batch mixing, batch average silhouette width (ASW) and principal-component-regression batch reduction. The biology-preservation domain comprised differential-abundance preservation, highly variable protein conservation, biology k-nearest-neighbour balanced accuracy, biology ASW, graph connectivity and isolated-label ASW.

The tested methods separated into distinct performance clusters (**Extended Data Fig. 4c,d**). The uncorrected, ComBat and quantile-normalized matrices retained the highest biology-preservation scores (0.744, 0.690 and 0.682, respectively), but achieved little batch correction (0.028, 0.032 and 0.000, respectively). In contrast, WaveICA, BERT-limma, TAMPOR and BERT-TAMPOR achieved high batch-correction scores (0.948–1.000) but lower biology-preservation scores (0.318–0.335). Within-metric ranks confirmed that no method dominated across all nine metrics (**Extended Data Fig. 4b**). Limma provided the most balanced correction, combining high batch-correction (0.915) with the highest biology-preservation among methods that achieved substantial batch removal (0.514), yielding the top composite score (0.674; **Extended Data Fig. 4c,d**).

Together, these results indicate that large-scale CSF matrix integration is feasible, but that batch correction imposes a measurable cost on preservation of biological structure. Limma represented the best batch–biology compromise in the shared 917-protein benchmark. Meta-program discovery was performed on study-wise protein matrices and did not use batch-corrected matrices. For atlas-wide sample-level program activities and protein-level analyses, we used limma- or BERT-limma-corrected matrices.

8.3 Supplementary Note 3. Relationship between the core CSF proteome and abundance-derived meta-programs

To determine how the detection-defined core CSF proteome related to the abundance-derived meta-program atlas, we compared the union of proteins assigned to the 13 consensus meta-programs with the Tier A/B core proteome and its six Core Classes (CC1–CC6). The two organizational layers showed substantial but incomplete overlap (**Extended Data Fig. 5a,b**). Of the 490 proteins with a non-redundant consensus meta-program assignment, 351 overlapped the 747-protein Tier A/B core, corresponding to 71.6% of meta-program proteins and 47.0% of core proteins. Meta-programs were enriched for higher detection tiers, with Tier A/B proteins

comprising the majority of MP1, MP2, MP3, and MP10 membership (**Extended Data Fig. 5c**), consistent with stably detected proteins contributing disproportionately to recurrent abundance structure. Lower-tier (C–E) proteins were concentrated in immune and barrier programs (MP4, MP7, MP12), reflecting their context-dependent appearance in CSF.

There was partial correspondence between the Core Class organization of the core proteome and the consensus meta-programs. Several biologically concordant reciprocal best matches were observed, including plasma/complement (CC1 and MP1) and ECM/matrisome (CC3 and MP3), whereas other Core Classes were distributed across multiple meta-programs (e.g., CC4 across MP13, MP2, MP5 and MP11) (**Extended Data Fig. 5d,e**). Together, these analyses indicate that the meta-program atlas remains grounded in the recurrent CSF core but captures an orthogonal organizational layer defined by cross-cohort abundance covariation rather than detection frequency alone.

8.4 Supplementary Note 4. Peripheral plasma ingress and frank blood signatures

Because CSF contains plasma-derived proteins, peripheral signal may reflect physiological influx, barrier dysfunction, or frank blood contamination. We therefore derived two orthogonal signatures: a liver-enriched, QAlb-correlated plasma-ingress signature and an erythrocyte-enriched frank-blood signature defined from bloody-versus-clean CSF contrasts (**Extended Data Fig. 7a**).

The 20-protein plasma-ingress signature was constructed by integrating three lines of evidence: (*i*) plasma-versus-CSF biofluid contrasts (Li 2021, Zhao 2018), (*ii*) liver-origin specificity from HPA and JensenLab expression atlases (τ and Gini indices; **Extended Data Fig. 7b–d)**, and (*iii*) QAlb correlation across the Bader 2026, Schmid 2021, and Held 2024 cohorts (**Extended Data Fig. 7e,f)**. A 10-protein frank-blood signature was separately derived from bloody-versus-clean CSF contrasts (Bader 2026, Riviere-Cazaux 2025, Mikolajewicz 2022; **Extended Data Fig. 7g)** intersected with *a priori* erythrocyte markers from HPA blood expression, PanglaoDB, and CellMarker 2.0 (**Supplementary Table 12**). Pathway enrichment confirmed the expected biological distinction between the two signatures (**Extended Data Fig. 7h**). Importantly, the plasma- and blood-derived scores were near-orthogonal (Pearson $r = 0.022$, 95% CI 0.005–0.040; $P = 0.014$; $n = 11{,}974$), establishing plasma ingress and frank blood as separable signals.

We projected both signatures onto meta-program activity to test whether peripheral signal was global or program-specific (**Extended Data Fig. 7i**). Plasma ingress was selectively enriched, strongest in MP1 (plasma/complement) with secondary coupling to MP10, MP7, MP6 and MP12 (complement-regulatory, barrier/epithelial, humoral immune and neutrophil programs), while neuronal/synaptic and lysosomal-catabolic programs showed weak or negative

associations. The frank-blood signature was more restricted, enriched in MP4 (myeloid stress/blood-associated inflammation) with limited association elsewhere.
